# A unique polygenic mouse model of obesity exhibits a distinct immunological profile that may offer protection against systemic inflammation, diabetes, and behavioral impairments

**DOI:** 10.3389/fimmu.2025.1601809

**Published:** 2025-09-12

**Authors:** Ulrike Gimsa, Dirk Koczan, Ellen Kanitz, Armin Tuchscherer, Alexander Rebl

**Affiliations:** ^1^ Research Institute for Farm Animal Biology (FBN), Dummerstorf, Germany; ^2^ Core Facility Genomics, Institute for Immunology, Rostock University Medical Centre, Rostock, Germany

**Keywords:** behavioral testing, cytokines, inflammation, microarray, polygenic obesity, MDSC

## Abstract

In both humans and mice, obesity is often associated with peripheral and central inflammation, which can lead to diabetes, dysregulation of the stress response, changes in affective behavior, and memory impairment. The DU6 polygenic mouse line was selected over more than 180 generations for a high body mass. Unlike other mouse lines, DU6 mice do not develop diabetes despite significant obesity. We performed a series of behavioral tests on male mice because obesity is often associated with cognitive and emotional impairment. DU6 mice showed no differences in spatial memory or anxiety compared to a control mouse line, based on performance in the Y-maze test, novel object recognition task, and elevated plus-maze test, whereas object memory was impaired in DU6 mice. After psychological stress evoked by the elevated plus-maze test, serum corticosterone concentrations were elevated only in the control mouse line, while corticosterone concentrations were already high in DU6 mice under non-stressed conditions. This elevation under control conditions was no longer detectable at an advanced age. We investigated the composition of immune cells in the spleen and assessed mitogen-induced T-cell activation *in vitro* in male DU6 mice. Compared to the control mouse line, DU6 mice exhibited significantly fewer CD4^+^ and CD8^+^ T cells, alongside a markedly higher proportion of macrophages and Gr-1^+^CD11b^+^ myeloid-derived suppressor cells. T-cell activation following mitogen stimulation was lower in DU6 mice than in the control mouse line. Following psychological stress induced by the elevated plus-maze test, the number of CD4+ T cells increased and the number of macrophages decreased in both mouse lines. The proinflammatory cytokines IL-1β, IL-6, and TNF-α were not detectable in the serum of male mice of both lines, ruling out systemic inflammation. Transcriptomic analysis also revealed no inflammation in the hippocampal tissue, but rather a distinct transcriptional signature in male DU6 mice compared to the controls. We propose that the high number of Gr-1^+^CD11b^+^ cells protects DU6 mice against systemic inflammation, diabetes, and behavioral impairment.

## 1 Introduction

According to the World Health Organization, 43% of adult humans were considered overweight with a body-mass index (BMI) ≥ 25, and 16% of the world population were considered obese with a BMI ≥ 30 in 2022 (1). In addition to BMI, other parameters are required to assess the nutritional status of an organism, such as waist circumference, body composition in terms of the ratio of muscle, fat, and bone, and metabolic changes (2).

In humans, obesity may be linked to metabolic syndrome and diabetes as well as dysregulated hypothalamus-pituitary-adrenal (HPA) axis, low-level systemic inflammation, and an impaired immune response (3–6). However, not all obese individuals develop metabolic complications. This phenomenon is referred to as metabolically healthy obesity (MHO), or metabolically benign obesity, although its definition remains inconsistent (7–10). MHO is mostly defined as having a BMI > 30 kg/m^2^, normal glucose and lipid metabolism parameters, and a normal cardiovascular fitness. The concept of MHO is controversial because MHO can be a temporary condition, as people who were originally metabolically healthy can develop cardiovascular disease within a few years (10–12). Animal models of obesity almost exclusively exhibit metabolic disease such as diabetes. Here we present the obese mouse line DU6, which is unique in not developing diabetes and could therefore be of interest for studying certain aspects of MHO.

Common animal models of obesity are also used to investigate the underlying mechanisms of comorbidities observed in humans, including cognitive dysfunction and psychological disorders [for review, see (13–16)]. The link between obesity and neuroinflammation, memory deficits and behavioral changes is the development of systemic inflammation. Obesity promotes infiltration of immune cells into adipose tissue and results in the secretion of proinflammatory cytokines and adipokines. These inflammatory mediators circulate in the bloodstream and induce peripheral inflammation as well as neuroinflammation. This state is exacerbated by obesity-induced gut dysbiosis, which induces a leaky intestinal barrier, thereby promoting neuroinflammation and neurodegeneration via the gut-brain axis (17). Systemic inflammation can lead to a spill of proinflammatory cytokines into the brain via a leaky blood-brain barrier or by transport proteins, activation of perivascular macrophages, which in consequence lead to an activation of microglia and astrocytes. Once activated, these brain immune cells produce inflammatory mediators and cytokines themselves (18). In a study on mice on a high-fat diet, microglial activation in the hippocampus along with cognitive impairment has been demonstrated (19). High-fat diet has also been shown to induce neuroinflammation in the hypothalamus, inducing autonomous system dysfunction (20).

Transgenic models of obesity mimic some of the physiological, psychological, and cognitive alterations seen in obese humans but are restricted to one gene, while human obesity is multicausal. Typical transgenic mouse models include the *ob/ob* mice, which do not produce leptin due to a mutated leptin gene (21), and the *db*/*db* mice, which do not express the leptin receptor (22, 23). *Ob/ob*, and *db/db* mice, show impaired spatial memory and increased anxiety associated with an altered activity of the HPA axis as well as systemic and central nervous system inflammation (24–27). While these transgenic models make it possible to elucidate the role of a specific gene in the complex phenomenon of obesity, the results obtained may not be readily transferable to the clinical context. Diet-induced obesity in rodents based on high-fat diets induces a number of physiological, cognitive, psychological, neuroendocrine, and immune alterations also seen in obese humans and mostly results in diabetes. However, it cannot be ruled out that these changes are a direct result of high dietary fat levels (28). Unlike transgenic mouse models and models with a high-fat diet, polygenic obesity rodent models have the advantage that obesity results from a genetic predisposition to obesity and overfeeding with standard feed, thus excluding the effects of dietary imbalances.

The long-term selection mouse line DU6 is characterized by obesity due to high feed intake of standard mouse chow, in addition to a genetically predisposed high feed efficiency. These mice do not develop diabetes (29) despite increased insulin and leptin levels (30, 31), which contrasts with other polygenic mouse models of obesity (32, 33). However, recent work has shown that life expectancy is reduced in these mice (31). Psychological, neuroendocrine, and immune responses in this mice model remain largely unexplored. This study aimed to further characterize the DU6 line, providing insights into obesity and its consequences in a unique polygenic model that dissociates obesity from diabetes. Given that systemic inflammation is a known link between obesity and diabetes, as well as obesity-related cognitive and affective impairment, it was of interest to examine whether inflammatory processes play a role in the DU6 mouse model. Because the DU6 mice, despite their pronounced obesity, do not develop diabetes, we set out to investigate if DU6 mice develop (i) cognitive and affective impairments associated with (ii) HPA axis dysregulation, (iii) immune alterations, and (iv) low-level systemic inflammation and neuroinflammation.

## 2 Materials and methods

### 2.1 Animals

**Figure 1:**
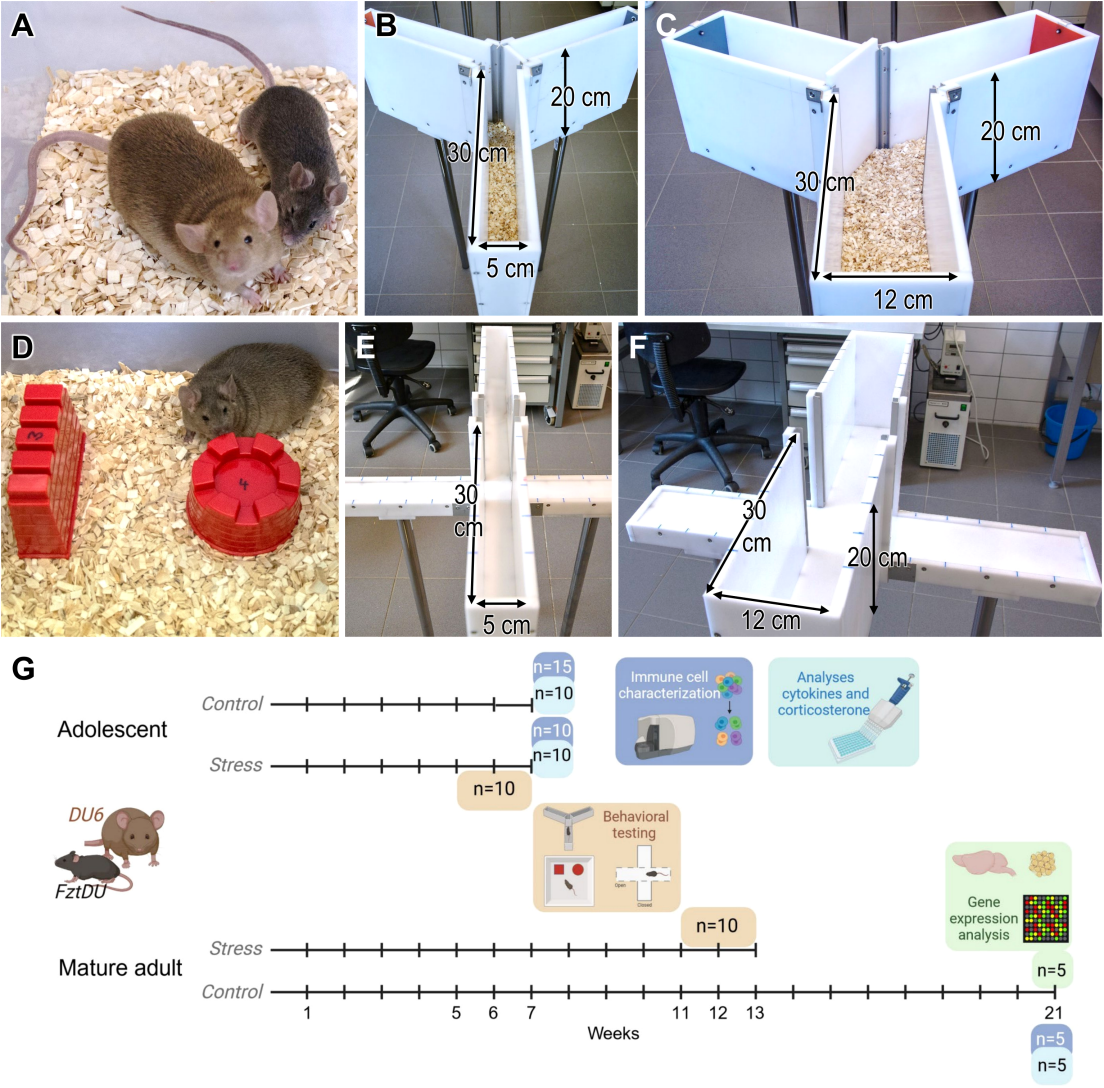
**(A)** Size example of two male DU6 (left) and FztDU (right) mice at 14 weeks of age. **(B)** Standard-size Y-maze with the left arm marked by a red plate (open) and the right arm marked by a gray plate (closed); **(C)** wider Y-maze with the left arm marked by a gray plate (closed) and the right arm marked by a red plate (open). Note that either the left or right arm of the larger and smaller Y-maze was closed or opened based on a randomized scheme. Arrows in **(B, C)** indicate the length, height and width of the passageways. **(D)** DU6 mouse approaching one of the two differently shaped objects, wall (left) and tower (right). **(E)** Standard-size and **(F)** wider EPM; arrows in **(E, F)** indicate the length, height and width of the passageways. **(G)** shows the experimental setup and the number of animals included in the depicted analyses. Each time line corresponds to one group of animals from each mouse line. Created in BioRender. Gimsa, U. (2025) https://BioRender.com/f80x0rn.

All the procedures were pre-approved by the local Animal Care Committee (LALLF 7221.3-1-006/18, date of approval: April 03, 2018) and conducted in compliance with the European Council Directive of 24 November 1986 (86/609/EEC). Only male mice of two mouse lines were used: (i) DU6, an outbred line subjected to 180 generations of selective breeding for high body mass at six weeks of age, and (ii) its non-selected control line FztDU (29, 30, 34–36) (**Figure 1A**). The outbred strain FztDU was bred from four outbred (NMRI, Han : NMRI, CFW, CF1) and four inbred strains (CBA, AB, C57BL, XVII) as described previously (30, 35). Both lines were bred and housed in the experimental animal facility of the FBN under specific pathogen-free conditions, at a controlled temperature of 22.5 ± 0.2°C, with at least 40% humidity and a 12 h:12 h light-dark cycle. Feed pellets (ssniff Spezialdiäten GmbH, Soest, Deutschland) and water were available *ad libitum*.

Mice were removed individually from their cages only for behavioral testing and were returned immediately afterward. The randomly selected test individuals were ten male mice from each of the FztDU and DU6 lines, which averaged 35.0 ± 2.9 g (FztDU; mean ± SD) or 91.8 ± 4.5 g (DU6) at seven weeks, and 46.7 ± 4.8 g (FztDU) or 122.8 ± 8.0 g (DU6) at 13 weeks of age (**Figure 1A**). Forty mice were used for behavioral experiments in total. The seven-week-old mice were euthanized within 10 minutes after the last behavioral test, and serum and spleens were isolated. These samples represented stress responses and were compared to those of age-matched controls (n = 10 in each mouse line). The mice were euthanized in a separate laboratory to avoid any influences of stress on the remaining mice. After cervical dislocation, the mice were rapidly decapitated, and their trunk blood was collected. Blood was allowed to clot for 2 h and then centrifuged at 2,000 × g for 10 min. Sera were stored at –20°C until corticosterone and cytokine analysis was performed. Spleens were isolated for flow cytometry and cell culture. Five additional mice per mouse line were euthanized at 21 weeks of age for gene expression studies, analysis of corticosterone concentration and characterization of immune cells. Hippocampi from these mice were isolated from the brains, snap-frozen in liquid nitrogen, and stored at –80°C until transcriptomic analysis was conducted. The experimental setup is summarized in **Figure 1G**.

### 2.2 Behavioral tests

#### 2.2.1 General remarks

Behavioral testing started with mice of both lines at the age of approximately five weeks (n = 10 per mouse line) or at approximately eleven weeks (n = 10 per mouse line), hereinafter referred to as the adolescent and the mature adult mouse cohorts. In the week before behavioral testing, mice became accustomed to handling by the person conducting the test over a two-day period. Bedding was provided in the test arenas and housing cages, except for the elevated plus-maze (EPM). Testing consisted of a sequence of three tests administered at one-week intervals: The Y-maze test for spatial memory and a novel object recognition (NOR) test were followed by the EPM test (**Figures 1B–F**). The EPM test was considered to induce the highest stress levels of all the tests. Consequently, we performed this test last to minimize any carry-over effects between the tests. Every test was conducted under 30-lux illumination to increase the activity of the mice and reduce their anxiety. Each test arena was cleaned of excrements between the individual behavioral tests to reduce the irritating odors of conspecifics. All experimental sessions were recorded using a Sony HDR-CX240E camera, and the video footage was analyzed with Observer XT 12 software (Noldus Information Technology BV, Wageningen, Netherlands).

#### 2.2.2 Y-maze test

The Y-maze test with forced choice alternation evaluates spatial orientation memory based on the natural exploratory behavior of the animals. Two sizes of Y-shaped mazes were used, each consisting of three arms positioned at 120° to one another, connected by a central area (**Figures 1B, C**). The walls of both mazes were 20 cm high, and the arms were 30 cm long. The arms in the standard-size maze were 5 cm wide (**Figure 1B**), while the arms of the wider maze were 12 cm wide (**Figure 1C**). To distinguish between arms, one was marked with a gray plate, the second with a red plate, and the third was unmarked. In the first trial, one arm of the Y-maze was blocked, and the test mouse was placed at the end of the starting arm, with its head facing the wall. After five minutes of exploring the two accessible arms, the mouse was returned to its housing cage. Thirty minutes later, the same mouse was reintroduced to the maze, where now all three arms were open. The timing began as soon as the mouse exited the starting arm with all four paws, and it had five minutes to explore the maze. The blocked arm’s position (left or right) was randomized for each experiment with ten test mice per line. In the first test, five mature adult FztDU mice were tested in the standard-size Y-maze, while the other five were tested in the wider maze. As there were no significant differences in mouse behavior between the standard-size and wider Y-mazes, the test with the adolescent FztDU mice was performed only in the standard-size maze. All DU6 mice were tested in the wider maze.

To determine whether mice from the two breeding lines preferred exploring the novel arm over the familiar ones, we measured the time spent in each and the number of entries, defined as entering with all four paws. For the statistical comparison with the novel arm, the number of entries into the familiar arms and the time spent there were each averaged and divided by two, as there were two familiar arms but only one novel arm. In the following, these averages are referred to as entries into the familiar arms and duration of exploration of the familiar arms, respectively.

#### 2.2.3 Novel object recognition test

The NOR test was used to evaluate object memory, specifically assessing whether the mice of the two lines equally recognized objects they encountered and explored the day before. The test box was constructed of gray plastic, measuring 30.5 cm × 38.0 cm at the base with 24 cm high walls. Cement-filled sand molds were used as objects in four distinct shapes: cylinder, pyramid, wall, and tower. Every object was uniform in material and color, and approximately equal in size (around 5 cm in height; see **Figure 1D**). The setup was chosen to allow us to analyze whether individuals from the two lines exhibited any laterality in exploring novel objects or a preference for one of the objects.

On day 1 (d1) of the test, individual mice were allowed to habituate to the test box without any objects for 20 min. On day 2 (d2), the training day, individual mice spent five minutes in the experimental box without any objects. General behaviors, such as running, digging, rearing, grooming, and inactivity, were recorded and analyzed during this period. Two identical objects were then placed in designated positions and remained with the individual mouse for a 10-minute exploration period (**Figure 1D**). After this time, the mouse was returned to its housing cage. Object shapes were varied between mice to minimize any bias toward a specific shape. On day 3 (d3), the actual test day, each mouse was reintroduced to the empty test box. After five minutes, the familiar object (from the previous day) and a novel object were positioned at marked locations. The mouse was then given 10 minutes to explore both objects before returning to its home cage. The positions of familiar and novel objects were alternated between animals to control for any side preference within the box. Video recordings were analyzed with regard to the exploration time, defined as the time spent with the mouse’s nose in direct contact with an object, which was measured for each object. The number of exploratory contacts was also counted. In addition, we determined the discrimination index (DI), which shows the ability of the mice to discriminate between the novel and the familiar object. It is calculated using the formula


$$DI=\left(t_{n}-\;t_{f}\right)/\left(t_{n}+\;t_{f}\right)$$

with t_n_, time spent exploring the novel object and t_f_, time spent with exploring the familiar object (37).

The DI can vary between +1 and -1, where a positive score indicates more time spent with the novel object, a negative score indicates more time spent with the familiar object, and a zero score indicates no preference.

#### 2.2.4 Elevated plus-maze test

The EPM tests the conflict between the animals’ natural urge to explore and their instinctive avoidance of open spaces. We used two EPMs of different sizes. Each maze had four arms arranged in a “plus symbol” shape and connected at a central point (**Figure 1E, F**). Two opposing arms were enclosed in opaque 20 cm high side and end walls, while the two open arms had a 0.5 cm rim to prevent falls. The arms were divided into 5 cm segments by markings. In both maze variants, the walls were 20 cm high, and the arms measured 30 cm in length, while arm widths were 5 cm for the standard-size maze (**Figure 1E**) and 12 cm for the wider maze (**Figure 1F**). Mice were placed individually in the maze center, facing an open arm. After five minutes of exploration, the mouse was returned to its cage, and the output of urine and feces was recorded. Using the Observer XT 12 software, we performed anxiety-based tests on the following parameters: (i) entries into open arms, (ii) time spent in open arms, and (iii) distance walked in open arms. To avoid the influence of general activities, we calculated the relative number of entries into open arms (= entries into open arms × 100%/[entries into open arms + entries into closed arms]), the relative time spent on the exploration of open arms (= time spent in open arms × 100%/300)) and the relative distance in open arms (= distance in open arms × 100%/[distance in open arms + distance in closed arms]) as proxies for the individual anxiety level. Locomotor activity was determined as (i) entries into closed arms; (ii) distance in closed arms. The conflict between approaching and avoiding risk, i.e., entering the “dangerous” open arms was judged from (i) entries into the central position, (ii) time in the central position, and (iii) head dips from a closed arm into an open arm.

### 2.3 Immune cell characterization by flow cytometry

Spleen tissue was cut into pieces and disrupted in 6 ml PBS using a gentleMACS dissociator (Miltenyi Biotec, Bergisch Gladbach, Germany). The suspension was passed through a 70 µm cell sieve to remove tissue debris and centrifuged at 1000 × *g* for 10 min at room temperature. The supernatant was discarded, and the cell pellet was resuspended in 400 µl of RPMI-1640 medium (PAN-Biotech, Aidenbach, Germany). This cell suspension was equally divided into four reaction vials (Sarstedt, Nümbrecht, Germany), with 100 µl per vial. Erythrocytes were lysed by adding 1000 µl Versalyse (Beckman Coulter, Krefeld, Germany), followed by gentle mixing and a 15-minute incubation in the dark at room temperature. The suspensions were then centrifuged at 150 × *g* for 5 minutes at room temperature and washed twice.

Cells for staining were resuspended in 500 µl of staining buffer (PBS containing 1% BSA and 2 mM EDTA) per two tubes, combined, and filtered through a 50 µm mesh. The final concentration was adjusted to 1 × 10^7^ cells/ml. For lymphocyte activation, cells from the other two tubes were resuspended in 1000 µl of RPMI-1640 culture medium, supplemented with 10% fetal calf serum (FCS, PAN-Biotech), 50 µg/ml gentamycin, 2 mM L-glutamine, and 50 µM 2-mercaptoethanol (all from PAN-Biotech). After combining the two cell suspensions, they were filtered through a 50 µm mesh and adjusted to a concentration of 4 × 10^6^ cells/ml. Stimulation was achieved with 2 µg/ml concanavalin A (ConA) for 24 h at 37°C in an incubator. All reagents were purchased from Sigma-Aldrich/Merck (Taufkirchen, Germany). The stimulated cells were centrifuged at 150 × *g* for 5 minutes at room temperature, the supernatants were discarded, and the pellets were resuspended in staining buffer.

For flow cytometric analysis, fresh cells were stained with panels of antibodies targeting: myeloid-derived suppressor cells (MDSCs; Gr-1^+^CD11b^+^), macrophages (Gr-1^-^ CD11b^+^), T-helper cells (CD4^+^ CD3^+^), cytotoxic T cells (CD8^+^CD3^+^), B cells (B220^+^). Stimulated cells were stained using antibodies specific for activated CD4^+^ and CD8^+^ T cells (CD25^+^CD4^+^, CD69^+^CD4^+^, CD25^+^CD8^+^, and CD69^+^CD8^+^). Samples in which MDSCs and macrophages were stained were also stained with the pan-leukocyte marker CD45.2. To rule out nonspecific staining, cells were stained with the respective isotype controls that were conjugated with the matching fluorochromes. Each staining reaction used 0.25 µg of antibody per 10^6^ cells in a total staining volume of 100 µl. Staining was performed for 30 min at 4°C. Then 900 µl of staining buffer was added and the tubes were centrifuges at 150 × *g* for 5 minutes at room temperature. The supernatants were discarded, and the washing step was repeated. After centrifugation, the pellets were resuspended in 500 µl staining buffer. All the antibodies were purchased from Biolegend (Amsterdam, The Netherlands). Details on antibody clones and conjugates are provided in **Supplementary Table S1**.

Samples were analyzed on a flow cytometer (Gallios 3-Laser Analyzer, Beckman Coulter). For samples stained to detect MDSCs and macrophages, gating was performed on CD45.2^+^ cells. In contrast, gating was applied to live lymphocytes based on forward and side scatter parameters for samples analyzed for T and B cells.

### 2.4 Corticosterone analysis

Serum corticosterone concentrations were measured in duplicate using a commercially available rat/mouse corticosterone ELISA kit (DEV 9922; Demeditec Diagnostics, Kiel, Germany) according to the manufacturer’s instructions. The antibody used in this assay exhibited a cross-reactivity of less than 1.1% with any potentially competing plasma steroids. The test sensitivity was 6.1 ng/ml, with intra-assay and interassay coefficients of variation of 7.3% and 8.2%, respectively.

### 2.5 Cytokine assays

Serum samples were tested for IL-1β using a murine IL-1β ELISA (BE 45111, IBL International, Hamburg, Germany), following the manufacturer’s instructions. The sensitivity of the assay was 1.2 pg/ml. The intra- and interassay coefficients of variance (CV) values were 4.7% and 5.7%, respectively.

Serum samples were tested for IL-6 using a murine IL-6 ELISA (BE 45061, IBL International) according to the manufacturer’s instructions. The sensitivity of the assay was 6.5 pg/ml and the intra- and interassay CV values were 5.0% and 8.9%, respectively.

Serum samples were tested for TNF-α using a murine TNF-α ELISA (BE 45291, IBL International) in line with the manufacturer’s instructions. The sensitivity of the assay was 3.7 pg/ml and the intra- and interassay CV values were 6.5% and 5.7%, respectively.

### 2.6 RNA isolation, microarray hybridization and data processing

RNA was extracted from the hippocampus of five 21-week-old mice per line (FztDU or DU6) using the RNeasy Plus Kit (Qiagen, Hilden, Germany). First, entire hippocampi were separately homogenized in liquid nitrogen using mortar and pestle after adding 600 μl RLT Plus buffer (RNeasy Plus Kit, Qiagen). Subsequent to thawing the ten samples, an extraction using phenol/chloroform/isoamyl alcohol (25:24:1, pH 6.6; Thermo Fisher Scientific, Waltham, MA, USA) was performed, followed by a DNA-removal step and the spin column clean-up in accordance with the manufacturer’s protocol (RNeasy Plus Kit). The ten RNA samples were quantified (Nanodrop 1000, Thermo Fisher Scientific) and diluted to a 70 ng/μl concentration. RNA integrity was tested using an Agilent RNA 6000 Nano Chip with a Bioanalyzer 2100 instrument (Agilent Technologies). All ten samples achieved RNA integrity numbers above 9.0.

Microarray hybridization was carried out using the Applied Biosystems Clariom S Assay Kit (formerly Affymetrix, Thermo Fisher Scientific) according to the GeneChip^R^ Whole Transcript Sense Target Labeling protocol (Affymetrix), as previously described (38). In brief, 200 ng of total RNA per sample was amplified and converted into strand-identical single-strand DNA that was subsequently hybridized to Clariom S Arrays (Mouse) for 16 h at 45°C in the GeneChip^R^ Hybridization Oven 645 (Affymetrix). The microarrays were washed and stained by the GeneChipFluidis Station 450 (Affymetrix) and then finally scanned using the GeneChip Scanner 3000 7G (Affymetrix) at a resolution of 0.7 microns.

### 2.7 Confirmation of differentially expressed genes by quantitative PCR

Quantitative PCR (qPCR) was performed using the LightCycler 96 System (Roche, Mannheim, Germany). RNA samples from the hippocampus (previously used for microarray hybridization), hypothalamus, and abdominal fat tissue from each mouse line (n = 5 per line) were individually reverse-transcribed into cDNA using the SensiFAST cDNA Synthesis Kit (Bioline/Meridian Bioscience). Then, qPCR reactions were performed in 12-μl volumes consisting of 6 μl SensiFAST SYBR No-ROX Mix (Bioline), 5 μl cDNA, and 1 μl of primers (sense and antisense). Sense and antisense primers for three selected target genes and three reference genes were designed using Pyrosequencing Assay Design Software (Biotage AB, Uppsala, Sweden) (**Table 1**). The target genes included *Mid1* (sense: 5’-GCGCTATGACAAATTGAAGCAAAA-3; antisense: 5’- CTTTTGGCTAAACTCATCCAAACT-3’), *Gjb4* (5’-TGGACCTGCCTCTGAGTACAC-3; 5’-CGCATTTATGGAGGGCACTGC-3’), and *Ccl19* (5’-AAGTCTTCTGCCAAGAACAAAGG-3; 5’-TGATGCTCTGTCCCAGACCTAA-3’). *Eef2*, *Rpl38* (39), and *Rplp0* (5’-GGCCCGAGAAGACCTCCTT-3; 5’-AATCTCCAGAGGCACCATTGA-3’) were used as internal normalizer genes.

**Table 1:** General behavior of male FztDU and DU6 mice during 5 min in the empty box of the NOR task on day 2. Data are shown as LS means ± SE.

Behavior	FztDU	DU6	p-value (Tukey-Kramer test)
Mature adult mice	n = 10	n = 10	
Rearing (#)	**29.90 ± 1.73**	**5.90 ± 0.77**	**<0.001**
Rearing (s)	**50.98 ± 5.77**	**8.83 ± 5.77**	**<0.001**
Digging (#)	**23.40 ± 1.53**	**9.00 ± 0.95**	**<0.001**
Digging (s)	**48.60 ± 8.96**	**15.80 ± 8.96**	**<0.05**
Grooming (#)	1.90 ± 0.44	1.50 ± 0.39	0.502
Grooming (s)	11.03 ± 2.95	4.62 ± 2.95	0.142
Running (s)	99.73 ± 9.25	82.15 ± 9.25	0.196
Inactivity (s)	**89.67 ± 12.28**	**188.61 ± 12.28**	**<0.001**
Adolescent mice	n = 10	n = 10	
Rearing (#)	**39.80 ± 1.99**	**17.60 ± 1.33**	**<0.001**
Rearing (s)	**56.55 ± 9.21**	**23.40 ± 9.21**	**<0.05**
Digging (#)	**31.60 ± 1.78**	**16.80 ± 1.30**	**<0.001**
Digging (s)	**52.35 ± 7.62**	**23.88 ± 7.62**	**<0.05**
Grooming (#)	4.70 ± 0.69	3.40 ± 0.58	0.168
Grooming (s)	9.33 ± 2.01	8.98 ± 2.01	0.902
Running (s)	79.51 ± 6.11	84.41 ± 6.11	0.578
Inactivity (s)	**102.25 ± 9.66**	**159.33 ± 9.66**	**<0.001**

#, number; s, duration (seconds). Bold numbers indicate significant differences between the mouse lines.

The qPCR was conducted in a LightCycler 96 instrument (Roche) with the following thermal cycling conditions: initial denaturation at 95°C for 5 minutes, followed by 40 cycles of denaturation at 95°C for 5 s, annealing at 60°C for 15 s, and elongation at 72°C for 15 s, with fluorescence measurements at 72°C for 10 s. No-template controls were included to monitor contamination. Amplicons ranging from 100 bp to 151 bp were visualized on agarose gels to confirm product size and quality. Raw qPCR data were processed using the LightCycler 96 v1.1.0.1320 software application. Melting curves were individually analyzed to verify the absence of nonspecific amplification. Only quantification cycle (Cq) values between 5 and 35 were considered for downstream analysis.

### 2.8 Statistical analyses

Statistical analyses were conducted using the SAS software application, version 9.4 (SAS Institute Inc., 2012, Cary, NC, USA).

Flow cytometry data and corticosterone concentrations were evaluated by two-way analyses of variance (ANOVA) using the MIXED procedure of SAS. ANOVA models for these traits comprised the fixed classification variables: line (FztDU, DU6) and treatment (control, stress) and the interactions line × treatment for the immune cell differentiation markers with and without stress. Time of sampling (five different points in time when groups of mice of similar age but of different lines, with or without prior stress, were euthanized) was included as a random effect. Another model included only control mice of two ages. This model comprised the fixed classification variables: line (FztDU, DU6) and treatment (control, stress), and age (7 weeks, 21 weeks) and the interactions line × treatment and line × treatment × age. All pairwise differences of the least-squares (LS) means were tested using the Tukey-Kramer procedure. Significance was defined as p < 0.05. The results are expressed as LS means ± standard error (SE).

Continuous behavioral data were evaluated using repeated measurement analyses of variance (ANOVA) of the MIXED procedure. Count data were analyzed with relevant Poisson models and the GLIMMIX procedure.

For the NOR test, the fixed factor “line” (“FztDU”, “DU6”) was applied to all recorded parameters in the open field test. In novel object test 1, fixed factors included “line” (“FztDU”, “DU6”), “side” (“left”, “right”), and “object type” (“wall”, “tower”, “pyramid”, “cylinder”), with the interactions line × side × object, line × object, line × side, and side × object calculated between these factors. For novel object test 2, the fixed factors line (“FztDU”, “DU6”) and familiarity (“familiar”, “novel”) were applied, with interactions calculated; repeated measurements for familiarity were managed using a Compound Symmetry covariance structure. In the Y-maze, the fixed factors included “line” (“FztDU”, “DU6”), “maze size” (“standard-size”, “wide”), and “familiarity” (“familiar”, “novel”), with repeated measurements (familiarity) accounted for by the Compound Symmetry covariance structure. The interaction line × maze size, line × familiarity and line × familiarity × maze size were integrated in the analysis. For the EPM test, the fixed factors line (“FztDU”, “DU6”) and “maze size” (“standard-size”, “wide”) and the interactions were integrated in the analysis. Pairwise multiple comparisons were performed for all behavioral tests using the Tukey-Kramer test. Effects and differences were considered significant when p < 0.05.

The probe cell intensity (CEL) files extracted from scanned microarray images were imported into the Transcriptome Analysis Console (TAC, version 4.0.3.14; Thermo Fisher Scientific). Data normalization was performed using the SST-RMA algorithm, and sequence annotation was achieved using the *Clariom_S_Mouse.r1.na36.mm10.a1.transcript.csv* file. Differentially expressed genes (DEGs) were identified, based on an absolute fold change > 2.0 and a false discovery rate (FDR) q < 0.05. TAC software was employed to generate principal component analysis (PCA) and hierarchical clustering of the expression data. Clustering was applied to both probe sets and CEL signal data. Cluster distances were calculated using the complete linkage method, with Euclidean distance as the similarity metric.

Functional analysis was conducted using the Ingenuity Pathway Analysis software (IPA, Qiagen). The expression analysis has been restricted to “nervous system”, “CNS cell lines”, “immune cell lines” and “macrophage cancer cell lines” to evaluate canonical pathways in the hippocampus, denoted in subsequent sections as italicized terms. The Benjamini-Hochberg procedure was applied for multiple testing correction, with a p-value threshold of ≤ 0.01 used as the cutoff to ensure robust and reliable identification of potentially modulated signaling pathways. Pathway activity was assessed using the z-score metric to determine activation (z > 1) or repression (z < -1). Statistical analysis of qPCR data was performed using the Student’s *t*-test in GraphPad Prism (version 10.4.1). The expression dataset has been submitted to the Gene Expression Omnibus (GEO) under accession code GSE280980.

Graphs were generated using Sigma Plot software 11.0 (Systat Software GmbH, Düsseldorf, Germany) and GraphPad Prism software 10.4.1 (San Diego, CA, USA).

## 3 Results

### 3.1 Spatial memory in the Y-maze is intact in DU6 mice

Taking the different sizes of the two mouse strains into account, we considered different Y-maze dimensions: wider ones for the DU6 mice and standard-size ones for the FztDU mice (**Figures 1B, C**). This introduced, however, the challenge of different testing conditions between the two setups. On the other hand, the use of the wider maze for both FztDU and DU6 mice could also influence the results, because the corridors for FztDU mice are significantly wider in relation to their body dimensions than for DU6 mice in relative terms. To address this problem, we tested five mature adult FztDU mice in mazes of both sizes. The results showed no significant differences in the behavior of FztDU mice between the wide and standard-size mazes. Consequently, all ten FztDU mice were combined into a single dataset for graphical representation. The raw data are provided in **Supplementary Table S2**.

**Figure 2:**
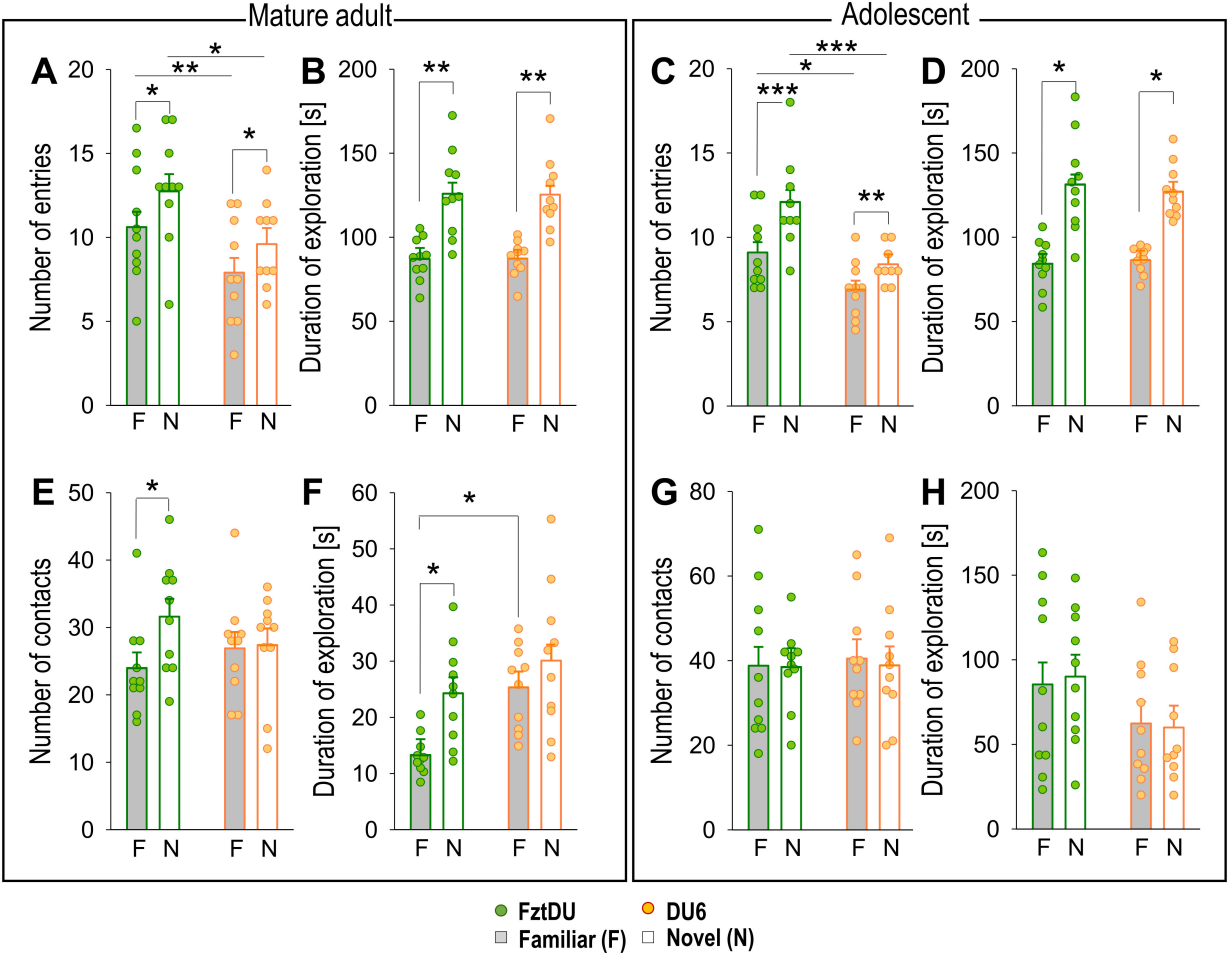
Spatial and object memory of mature adult and adolescent male FztDU and DU6 mice. The bar graphs compare exploratory behavior between two groups (FztDU, green; DU6, red) for mature adults (left panel) and adolescents (right panel). Data are shown as means ± SE for **(A, C)** number of entries into familiar arms (gray bars) versus novel arms (white bars) of the Y-maze, **(B, D)** duration of exploration of the Y-maze, **(E, G)** the number of contacts with a familiar object (gray bars) versus a novel object (white bars) in the NOR task, and **(F, H)** duration of exploration contacts with these objects in the NOR task. Statistical significance is indicated as follows: *p < 0.05, **p < 0.01, ***p < 0.001 (n=10 per mouse line; Tukey-Kramer test).

The number of entries into familiar arms or the novel arm, as well as the period of time that the mature adult FztDU mice spent in one of the two familiar arms or the novel arm did not differ significantly (p > 0.05) between the two maze sizes. Importantly, both DU6 and FztDU mice entered the novel arm more frequently than the familiar arms (p < 0.05; **Figure 2A**) and explored the novel arm significantly longer than the familiar ones (p < 0.05; **Figure 2B**). Notably, FztDU mice entered both the familiar and novel arms more frequently than DU6 mice (p < 0.01 for the familiar arms; p < 0.05 for the novel arms).

We also tested adolescent mice to match the age of the mice used in immune cell population analyses. Adolescent mice of both strains displayed the same preference for the novel arm over the familiar arm, entering the novel arms more frequently than the familiar ones (p < 0.01 in DU6 mice, p < 0.001 in FztDU mice; **Figure 2C**) and spending significantly more time in the novel arm (p < 0.01 in both lines; **Figure 2D**). Comparable to the mature adult mice, adolescent FztDU mice entered the familiar and the novel arms more often than the DU6 mice did (p < 0.05 for the familiar arms; p < 0.001 for the novel arms).

### 3.2 The behavior of DU6 mice in the NOR test differs from that of FztDU mice

#### 3.2.1 DU6 Mice are less active than FztDU mice

The training phase of the NOR task on day 2 allowed the general behavior of both mice lines to be studied in the empty experimental box, which lacked any objects to explore. The adolescent DU6 mice were generally less active than adolescent FztDU mice. The FztDU mice reared more often and longer than their DU6 counterparts in the 5-minute testing period. Concomitantly, FztDU mice dug more often and longer than DU6 mice (**Table 1**). There were no line differences in duration, the frequency of grooming, or the running duration. However, FztDU mice showed a shorter duration of inactivity than DU6 mice (**Table 1**). In the mature adult mice cohort, the line-dependent effects observed were qualitatively similar, although even more pronounced (**Table 1**).

#### 3.2.2 Object memory is impaired in DU6 mice

The second phase of the NOR test on the second day of testing focused on the general curiosity of mice from both lines towards novel objects and served the acquisition of object memory (**Figure 1D**).

We observed that the cohort of mature adult DU6 and FztDU mice preferred neither two novel objects nor the location of a particular object (data not shown). The objects were exchanged between mice to exclude any bias.

In the test phase of the NOR task on day 3, the cohort of mature adult FztDU mice made more and longer contact with a novel object than with the familiar object (known from the previous testing day) with p < 0.05. In contrast, mature adult DU6 mice approached both objects in a similar manner (**Figures 2E, F**), indicating an insufficient object memory. Apparently, this was not due to differences in locomotor behavior or motivation, as DU6 mice made a similar number of contacts to both familiar and novel objects as FztDU mice but spent more time exploring the familiar objects (p = 0.034). The same result was obtained by the DI, which is a relative measure that also takes into account variations in the animals’ overall activity. FztDU mice showed a higher DI (DI = 0.273 ± 0.053) than DU6 mice (DI = 0.059 ± 0.053; p < 0.05; see **Supplementary Figure S2**). The low DI in DU6 mice indicates a deficit in object memory.

To exclude the effects of aging on object memory, we tested adolescent mice of both lines. Surprisingly, the cohort of adolescent mice showed unexpected behavior. Based on the number of contacts and the duration of contact, neither line of mice was able to distinguish the novel object from the familiar object (**Figures 2G, H**). To rule out the possibility that the decisive differences in the examination of the new versus the familiar object occurred in the first minutes of the 10-minute test phase, we analyzed the 10 minutes individually (**Supplementary Figure S1**). But even so, no differences between the examination of the new and familiar objects were detectable in either FztDU or DU6 mice. The discrimination was close to zero for both FztDU (DI = 0.056 ± 0.106) and DU6 mice (DI = -0.009 ± 0.106), indicating that there was no preference for the novel object over the familiar one (**Supplementary Figure S2**).

### 3.3 DU6 mice do not display increased anxiety-like behavior

To ensure comparable test conditions for the two mouse strains, which differ significantly in size, we considered whether to test the FztDu in a maze with the same dimensions as the one used for DU6 mice. However, we had to account for the fact that the FztDU would have more lateral space in the wider maze (**Figure 1F**) than the DU6 mice in the same maze. As for the Y-maze, we, therefore, first compared mature adult mice to determine whether the additional space that FztDU mice have in the wider maze, compared to the standard-size maze (**Figure 1E**), influences their behavior. Indeed, there were differences: the FztDU mice in the wider maze entered the open arms more frequently than they did in the standard-size maze. Also, they spent less time in the center between the arms and performed fewer head dips into the open arms. The relative frequency of entering the open arms and the relative distance traveled in the open arms did not differ between the differently sized EPMs (**Table 2**). We conclude that the additional lateral space that the FztDU mice have in the large EPM reduces their reluctance to enter the open arms, as the width of the open arms does not pose a risk of falling.

**Table 2:** EPM behavior of male FztDU and DU6 mice. Mature adult FztDU mice were tested in the standard-size and wider mazes. Data are shown as LS means ± SE.

Behavior	FztDU (standard-size maze)	FztDU (wider maze)	DU6 (wider maze)	p-value (Tukey-Kramer test) FztDU (standard-size maze) vs. DU6 (wider maze)	p-value (Tukey-Kramer test) FztDU (wider maze) vs. DU6 (wider maze)	p-value (Tukey-Kramer test) FztDU (standard-size maze) vs. FztDU (wider maze)
Mature adult mice	n = 5	n = 5	n = 10			
Anxiety-like behavior						
Open arm entries (#)	**6.60 ± 1.15**	**12.00 ± 1.55**	6.40 ± 0.80	0.989	**<0.01**	**<0.05**
Time in open arms (s)	57.83 ± 21.55	116.57 ± 21.55	63.72 ± 15.24	0.973	0.142	0.161
Distance in open arms (cm)	269.00 ± 106.57	550.00 ± 106.57	256.50 ± 75.36	0.995	0.091	0.179
% Open arm entries	31.04 ± 7.59	44.97 ± 7.59	35.68 ± 5.37	0.873	0.587	0.415
% Time in open arms	19.28 ± 7.18	38.86 ± 7.18	21.24 ± 5.08	0.973	0.142	0.161
% Distance in open arms	31.24 ± 8.24	45.55 ± 8.24	29.76 ± 5.83	0.988	0.993	0.453
Locomotor behavior						
Closed arm entries (#)	**14.60 ± 1.71**	14.00 ± 1.67	**7.60 ± 0.87**	**<0.01**	**<0.01**	0.966
Distance in closed arms (cm)	569.00 ± 59.65	625.00 ± 59.65	417.00 ± 42.18	0.124	**<0.05**	0.787
Approach/avoid conflict						
Central position entries (#)	**20.60 ± 2.03**	25.80 ± 2.27	**13.80 ± 1.17**	**<0.05**	**<0.001**	0.233
Time in central position (s)	**136.40 ± 12.17**	**68.03 ± 12.17**	**69.77± 8.61**	**<0.001**	0.993	**<0.01**
Head dips	**25.60 ± 2.26**	**15.00 ± 1.73**	**14.80 ± 1.22**	**<0.001**	0.317	**<0.01**
Adolescent mice	n = 10		n = 10			
Anxiety-like behavior						
Open arm entries (#)	**12.90 ± 1.14**		**6.30 ± 0.79**	**<0.001**		
Time in open arms (s)	96.01 ± 17.93		94.17 ± 17.93	0.943		
Distance in open arms (cm)	512.00 ± 86.73		290.00 ± 86.73	0.087		
% Open arm entries	40.28 ± 6.08		38.05 ± 6.08	0.798		
% Time in open arms	32.00 ± 5.98		31.39 ± 5.98	0.943		
% Distance in open arms	40.61 ± 6.37		35.19 ± 6.37	0.555		
Locomotor behavior						
Closed arm entries (#)	**16.90 ± 1.30**		**7.60 ± 0.87**	**<0.001**		
Distance in closed arms (cm)	**652.50 ± 42.86**		**424.50 ± 42.86**	**<0.01**		
Approach/avoid conflict						
Central position entries (#)	**30.50 ± 1.75**		**14.40 ± 1.20**	**<0.001**		
Time in central position (s)	122.60 **±** 13.66		87.00 **±** 13.66	0.082		
Head dips (#)	**26.40 ± 1.62**		**14.60 ± 1.21**	**<0.001**		

#, number; s, duration (seconds). Bold numbers indicate significant differences between the mouse lines.

Assuming that comparable spatial conditions in the EPM are necessary for valid comparisons, we opted to test adolescent FztDU in the standard-size maze while DU6 mice were tested in the wider one. Under these conditions, DU6 mice did not exhibit more anxiety than FztDU mice; they entered the open arms just as often in relative terms, spent the same amount of time on the open arms in both relative and absolute terms, and covered the same distances there. This applies to adolescent and mature adult mice to the same extent. Only the absolute number of open-arm entries differs between the strains in adolescent mice, but not in mature adult mice (**Table 2**).

**Figure 3:**
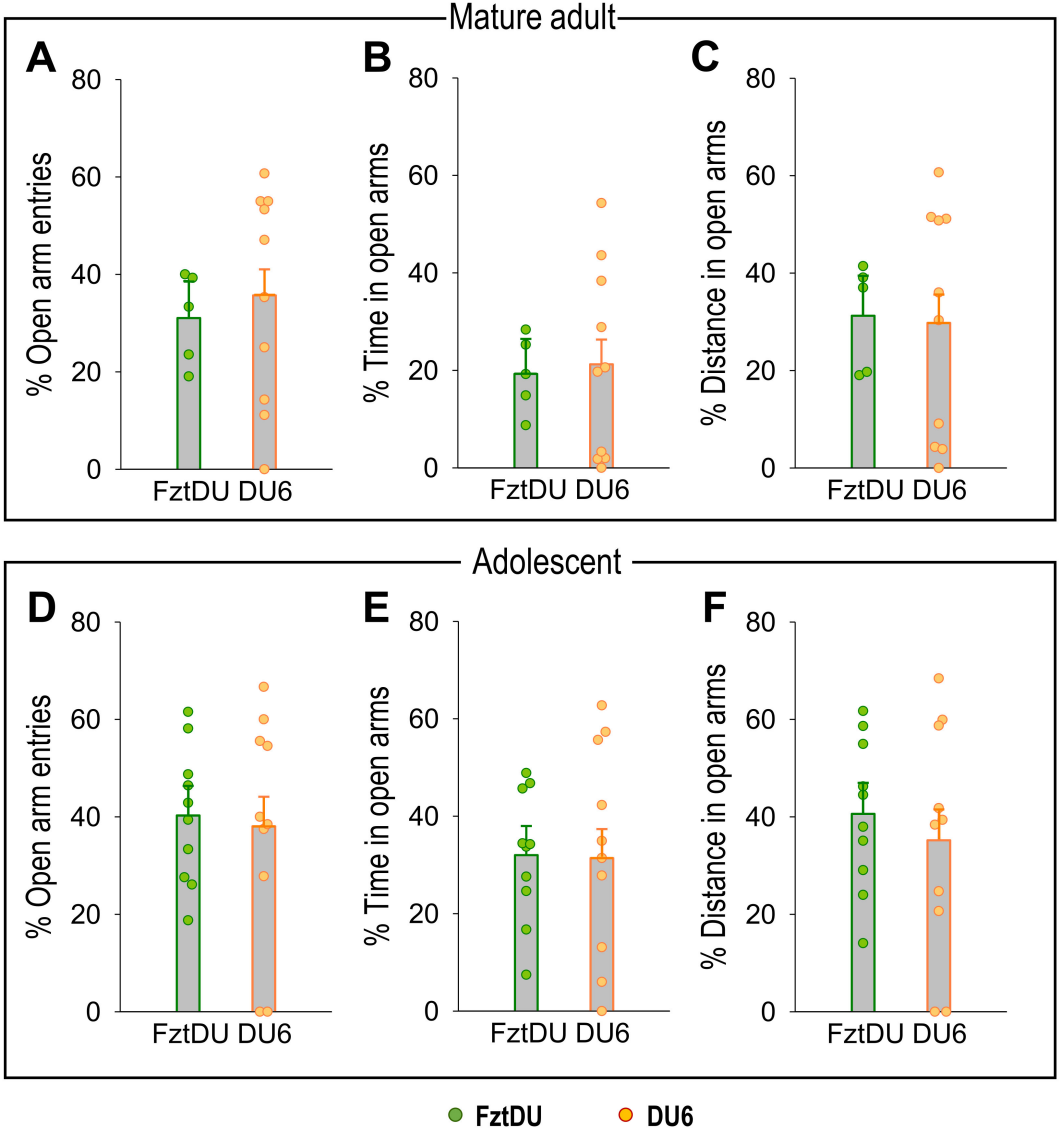
Anxiety-related behavior in the EPM for mature adult and adolescent male FztDU and DU6 mice. The graphs show the percentage of **(A, D)** open arm entries, **(B, E)** time spent in open arms, and **(C, F)** distance traveled in open arms for the two mouse lines FztDU (green) and DU6 (red). Data are presented separately for mature adult mice [upper panel: **(A–C)**] and adolescent mice [lower panel: **(D–F)**]. Data points represent individual mice, and bars represent the LS means and SE (n=10 per mouse line; Tukey-Kramer test).

**Figure 3** shows the individual anxiety-related behavior of adolescent and mature adult DU6 mice in the wider EPM and FztDU mice in the standard-size EPM.

### 3.4 DU6 mice show permanently elevated plasma corticosterone concentrations in adolescence

**Figure 4:**
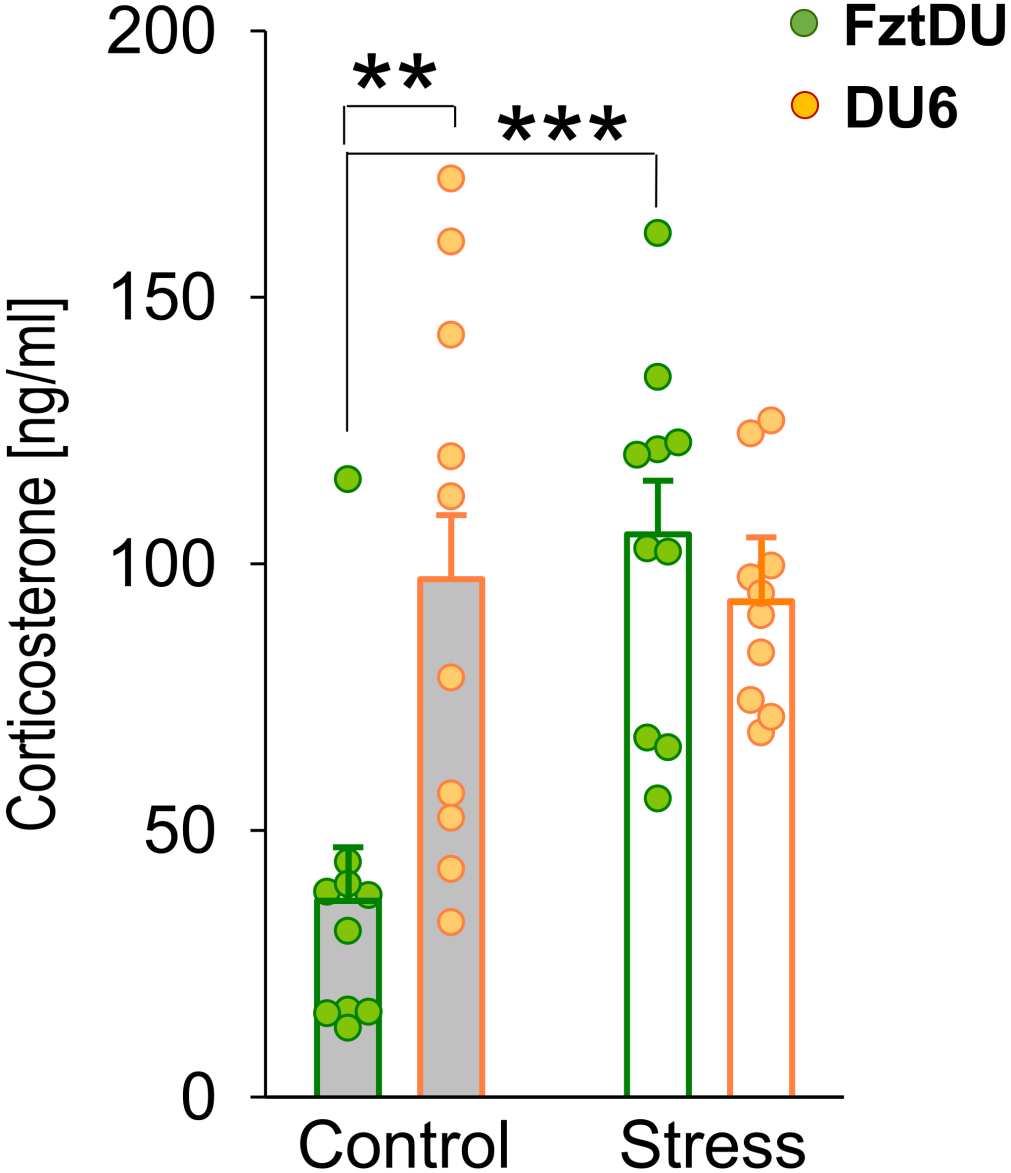
Corticosterone levels [ng/mL] in male FztDU (green bars) and DU6 mice (orange bars) under control and stress conditions. Bars represent the LS means and SE, with individual data points shown (**p < 0.01; ***p < 0.001; Tukey-Kramer test).

Our previous studies have established that EPM is a moderate psychological stressor for mice, inducing the release of corticosterone (40, 41). In this study, the EPM at 7 weeks of age more than doubled corticosterone levels of FztDU mice (FztDU control; means ± SE: 36.77 ± 10.08 ng/ml; stress (EPM): 105.52 ± 10.08 ng/ml; p < 0.001) (**Figure 4**). In contrast, corticosterone concentration in DU6 mice remained unchanged (DU6 control: 97.14 ± 11.98 ng/ml; stress (EPM): 93.01 ± 11.98 ng/ml). One thing worthy of note was that their baseline corticosterone levels were already more than twice as high as those of FztDU mice, and the moderate EPM-induced stress did not further upregulate corticosterone levels in DU6 mice. Interestingly, control DU6 mice do not show significantly elevated serum corticosterone concentrations compared to FztDU mice at 21 weeks of age (DU6: 43.17 ± 16.95 ng/ml; FztDU: 36.02 ± 14.26 ng/ml). The corticosterone concentrations of control DU6 mice at 21 weeks of age did not differ significantly from those of control DU6 mice or from those of stressed DU6 mice at 7 weeks of age.

### 3.5 DU6 mice display altered immune cell populations and reduced T-cell activation when exposed to mitogen

**Figure 5:**
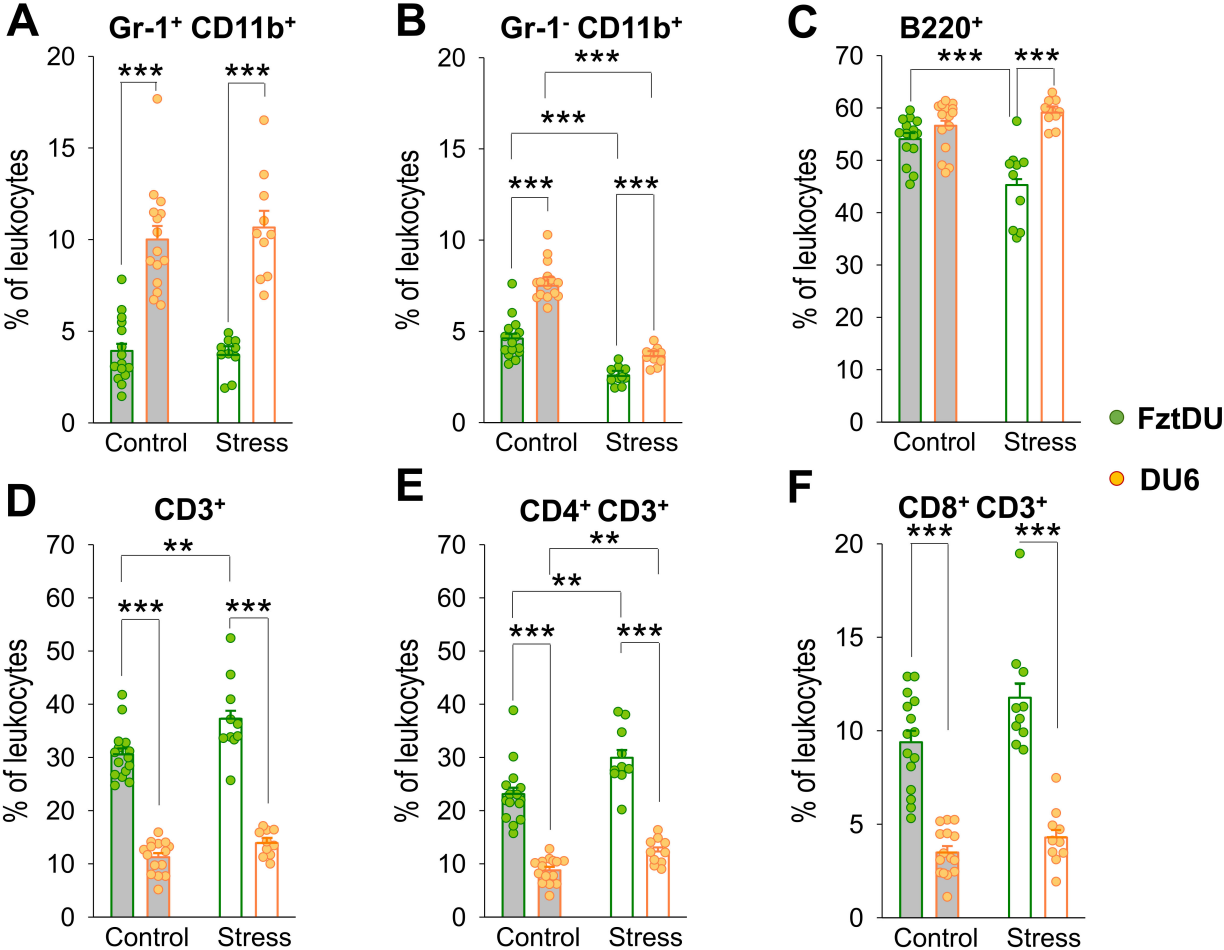
Leukocyte composition in male FztDU (green dots) and DU6 (orange dots) mice under control and stress conditions. Flow cytometric analysis was used to determine the proportion of **(A)** Gr-1^+^ CD11b^+^ cells, **(B)** Gr-1^-^ CD11b^+^ cells, **(C)** B220^+^ B cells, **(D)** CD3^+^ T cells, **(E)** CD4^+^ CD3^+^ T cells, and **(F)** CD8^+^ CD3^+^ T cells. Statistical significance is indicated as follows: **p < 0.01; ***p < 0.001. Data points represent individual mice, and bars represent the LS means and SE.

Our flow cytometric analysis of splenic leukocytes from both mouse lines revealed a significantly higher percentage of Gr-1^+^CD11b^+^ myeloid-derived suppressor cells (MDSCs) in DU6 compared to FztDU mice (**Figure 5A**). Similarly, the Gr-1^-^CD11b^+^ macrophage population was significantly larger in DU6 mice than in their FztDU counterparts (**Figure 5B**). B220^+^ B cell frequencies are similar between lines in the control condition (**Figure 5C**), whereas the relative proportion of T cells was lower in DU6 mice than in FztDU mice (**Figures 5D–F**).

DU6 mice exposed to acute stress by the EPM displayed a reduced proportion of macrophages in the spleen, while the other leukocyte populations examined did not show significant changes. In contrast, acute stress resulted in a decreased proportion of B220^+^ B cells in FztDU mice but not in DU6 mice (**Figure 5C**) and an increased proportion of CD3^+^ T cells in FztDU mice, as reflected by a higher percentage of CD4^+^ T cells (**Figures 5D, E**) but also a reduced proportion of macrophages (**Figure 5B**) in both lines. With increasing age, the proportion of macrophages decreases in both FztDu and DU6 mice. Otherwise, the immune cell populations remain unchanged (**Supplementary Table S3**).

**Figure 6:**
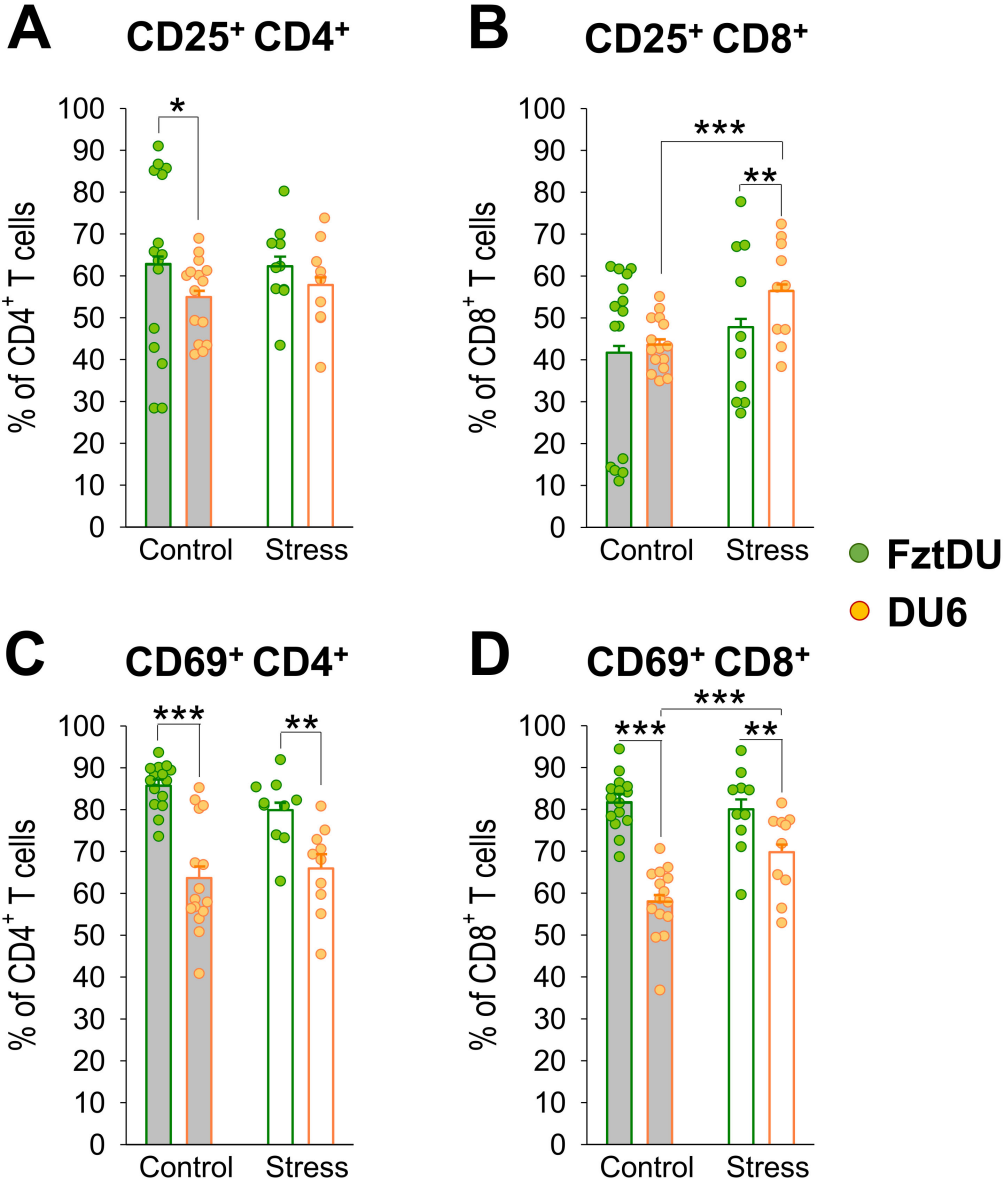
Activation of CD4^+^- and CD8^+^ T cells in male FztDU (green dots) and DU6 mice (orange dots) under control and stress conditions following stimulation with ConA. Flow cytometry was used to determine the proportions of **(A, C)** CD4^+^ T cells and **(B, D)** CD8^+^ T cells expressing activation markers, either **(A, B)** CD25^+^ or **(C, D)** CD69^+^. Statistical significance is indicated as follows: *p < 0.05; **p < 0.01; ***p < 0.001 (Tukey-Kramer test). Data points represent individual mice, and bars represent the LS means and SE.

To assess whether T-cell activation is different between the two mouse lines in adolescence, we stimulated lymphocytes with the T-cell mitogen ConA and then stained them for the T-cell activation markers CD25 and CD69. The proportion of CD25-expressing CD4^+^ cells among all CD4^+^ cells was lower in DU6 than in FztDU mice following stimulation with ConA (**Figure 6A**). The pattern for CD69 expression showed differences: after ConA stimulation, both CD4^+^ and CD8^+^ cell populations from DU6 mice displayed a lower proportion of CD69^+^ cells than those from FztDU mice (**Figures 6C, D**).

Acute stress increased the proportion of CD25-expressing CD8^+^ cells in response to ConA stimulation in DU6 mice. Thus, DU6 mice displayed a higher proportion of CD25^+^CD8^+^ cells than FztDU mice after stress (**Figure 6B**). However, the actual number of CD25 expressing CD8^+^ cells did not increase due to stress while the number of CD25-negative CD8^+^ cells decreased (**Supplementary Table S4**). Similarly, the proportion of CD69-expressing cells increased in DU6 CD8^+^ cells after stress but was still lower than in FztDU mice (**Figure 6D**). However, the actual number of activated CD4^+^ and CD8^+^ cells in response to ConA stimulation was lower in DU6 mice than in FztDU mice (**Supplementary Table S4**). The increase in the proportion CD69-expressing CD8^+^ cells among CD8^+^ cells due to stress resulted from an unchanged number of CD69^+^CD8^+^ cells with a concomitant decrease in CD69^-^CD8^+^ cells in DU6 mice (**Supplementary Table S4**).

### 3.6 Peripheral inflammation is not detectable in DU6 mice

ELISA was used to quantify the concentrations of the proinflammatory cytokines TNF-α, IL-1β and IL-6 in the serum of both mice lines. The protein levels of all the cytokines selected were below the detection limit in adolescent and 21 week-old mice of both lines with the exception of one 21 week-old DU6 mouse who had 40.66 pg/ml IL-6 in the serum.

### 3.7 Transcriptomic profiling reveals a distinct signature in DU6 mice

**Figure 7:**
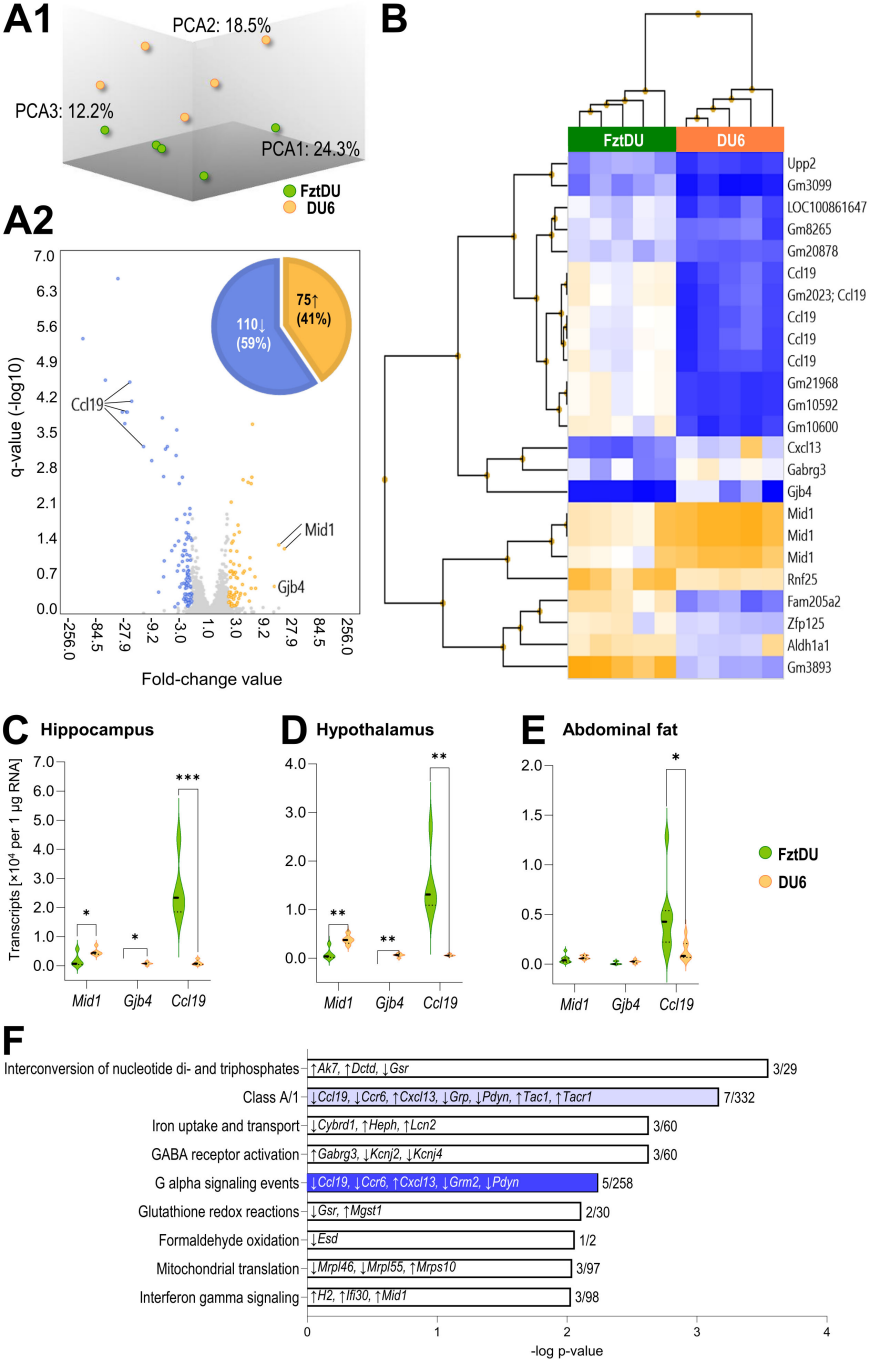
Microarray-based analysis of gene expression in hippocampus of male DU6 and FztDU mice. **(A1)** Principal Component Analysis (PCA) mapping the gene expression in DU6 (orange spheres) and FztDU (green spheres) mice (n = 5 per line). Note that the first three principal components (PCA1, PCA2, and PCA3) account for 55% of the variance in the dataset indicating a moderate separation of both datasets. **(A2)** Volcano plot illustrating the differentially expressed genes between DU6 and FztDU. Fold change (FC) values are shown on the abscissa, and the negative logarithm of q-values is displayed on the ordinate. Annotated genes with an absolute FC >10, including *Ccl19*, *Mid1*, and *Gjb4*, are labeled. The accompanying pie chart summarizes the distribution of genes higher (orange) and lower (blue) expressed in DU6 versus FztDU mice. **(B)** Heatmap with hierarchical clustering of DEGs with an absolute FC > 5 between DU6 and FztDU. Rows represent gene symbols and Gm features; columns correspond to individual samples. Expression levels are color-coded, with blue indicating lower gene expression and orange a higher expression. **(C–E)** Validation of the transcript abundance of selected genes in **(C)** the hippocampus, **(D)** hypothalamus, and **(E)** abdominal fat tissue of DU6 and FztDU mouse strains. Violin plots represent the distribution of normalized transcript levels (per 1 µg of RNA) for *Mid1*, *Gjb4*, and *Ccl19* in DU6 (orange bars) and FztDU (green). Individual data points are represented by dots within the violins. Statistical significance was assessed using Student’s t-test (*p < 0.05; ***p < 0.01; ***p < 0.001). **(F)** Significantly enriched canonical pathways (q < 0.01) in DU6 mice compared to FztDU mice. Pathways are listed on the ordinate, with bar lengths representing the negative log of the p-value. Each bar displays the genes upregulated (↑) or downregulated (↓) within the respective pathway. The ratio of regulated genes to the total number of genes in the pathway is indicated at the end of each bar. Light blue and dark blue bars represent pathways with negative z-scores. Created in BioRender. Gimsa, U. (2025) https://BioRender.com/f80x0rn.

Using microarray technology, we analyzed transcriptional patterns in the hippocampus of 11-week-old DU6 and FztDU mice. The comparison of resulting transcriptomes revealed 75 genes with higher transcript levels (41%) and 110 genes with lower transcript levels (59%) in DU6 mice compared to FztDU individuals, with an absolute fold change of > 2 and a q-value of < 0.05 (**Figures 7A1, A2**). Among these, 15 of the higher-expressed genes and 31 of the lower-expressed genes were identified as features with a ‘Gm’ prefix, indicating that they were not assigned to a canonical gene symbol but represent, for instance, pseudogenes or antisense transcripts. In DU6 mice, the highest transcript levels were observed for *Mid1* (midline-1; 12.7- to 15.9-fold increase) and *Gjb4* (gap junction protein beta 4; 10.9-fold increase) compared to FztDU mice. Conversely, the *Ccl19* gene (cc-motif chemokine ligand-19) was the most strongly expressed gene in FztDU mice, with a -3.2- to -22.2-fold change relative to the DU6 line (**Figures 7A2, B**).

Additionally, genes such as *Gabrg3* (gamma-aminobutyric acid A receptor, subunit gamma 3) and *Cxcl13* (chemokine ligand 13) exhibited at least a five-fold increased expression in DU6 versus FztDU mice, while *Upp2* (uridine phosphorylase 2), *Zfp125* (zinc finger protein 125), *Rnf25* (ring finger protein 25), and *Aldh1a1* (aldehyde dehydrogenase family 1, subfamily A1) showed similarly elevated expression in FztDU mice compared to their DU6 conspecifics (**Figure 7B**). A targeted search of the expression values for distinct markers including *Cd11b* and *Cd68* (encoding surface markers of macrophages/microglia), *Gfap* (encoding a marker for astrocyte activation), and *Nr3c1* (encoding the glucocorticoid receptor) and *Tnf*, *Il1b*, *Il6, and Il4, Il10 and Tgfb* (encoding the proinflammatory cytokines TNF-α, IL-1β and IL-6 and the antiinflammatory cytokines IL-4 and IL-10 and TGF-β) confirmed similar regulation in the hippocampus of DU6 and FztDU mice and did not indicate neuroinflammation or antiinflammatory activity (**Supplementary Table S5**).

The expression levels of the three most prominently differentially regulated genes between mouse lines (*Mid1*, *Gjb4, Ccl19*) were quantified by qPCR in hippocampal samples (**Figure 7C**), confirming the significant differences between the two mouse lines observed in the microarray analysis. A similar expression pattern was also detected in the hypothalamus for these three genes (**Figure 7D**). In abdominal fat tissue, a significantly different expression was detectable only for *Ccl19* (**Figure 7E**).

The identified differentially regulated genes were assigned to nine pathways, with a corrected p-value of less than 0.01 (**Figure 7F**). These pathways contribute to energy supply and metabolism (*glutathione redox reactions*, *formaldehyde oxidation*, *iron uptake and transport*, *interconversion of nucleotide di- and triphosphates*, *and mitochondrial translation*), sensory processing and signal transmission (*class A/1 [rhodopsin-like receptors*], *GABA receptor activation*, *G alpha signaling events*), and immunity (*interferon gamma signaling*). As expected, some of the most strongly regulated genes in DU6 mice are also involved in the pathways mentioned above, including *Ccl19* and *Cxcl13* (*class A/1* and *G alpha signaling events*), *Mid1* (*interferon gamma signaling*), and *Gabrg3* (*GABA receptor activation*).

## 4 Discussion

The present study focuses on the DU6 mouse line as a polygenic model for obesity. While both male and female DU6 mice are obese, we focused on male mice in this study. Obesity is associated not only with physiological changes but also with behavioral and emotional state alterations. Research in rodent models (20–22, 33) and studies in humans (13, 14, 42–45) have linked obesity to impaired cognitive function and increased anxiety. We therefore investigated spatial and object memory as well as anxiety-like behavior in DU6 mice compared to FztDU mice. The Y-maze provides insights into the spatial orientation of test mice, while the NOR task is used to assess object memory. While the Y-maze is typically considered a hippocampus-dependent task, the NOR task is considered at least partially hippocampus-independent (46–48). A study in rats demonstrated that the lateral entorhinal cortex, a crucial afferent of the hippocampus, plays a vital role in novel object recognition (49). Additionally, the EPM was used to assess the conflict between exploratory drive and the innate fear of open spaces, a behavior influenced by hippocampal function (50).

Behavioral testing using the Y-maze with an inter-test interval of 30 min revealed that DU6 mice possess intact spatial memory. In contrast, other studies in mice have demonstrated impaired spatial memory in the Y-maze (27, 28, 51). Both Dinel et al. and Andre et al. used an inter-trial interval of 30 min, as we did (27, 28), while a study in rats showed impaired spatial memory in the radial arm maze but not the Y-maze in a test with a 4-h inter-trial interval (52). All of these studies found signs of hippocampal inflammation, such as microgliosis and elevated levels of proinflammatory cytokines, which the authors attributed to cognitive decline. This may explain our deviating data, as DU6 mice did not show any sign of systemic or neuroinflammation.

Despite the absence of inflammation, recognition memory was impaired in mature adult DU6 mice, as determined by the NOR test. This test is based on the behavioral tendency of rodents to seek out and explore novel objects. Depending on the inter-test interval, either the short-term memory is tested for retention times of up to 1 h or the long-term memory after 24 h. Impaired object recognition memory was also found in other rodent studies. A study of diet-induced obesity in rats found impaired object memory after 1 h retention time (52). Studies of monogenetic obesity in *db*/*db* mice either found impaired object memory after 30 min retention time (27) or did not find any after 30 min or 24 h retention time (51). In contrast, a study of monogenetic obesity in melanocortin-4 receptor-deficient mice found impaired object memory after 24 h but not after 1 h retention time (27).

Surprisingly, adolescent mice of both FztDU and DU6 mouse lines did not successfully complete the NOR task. It is possible that the mouse lines we used were too developmentally immature at this age to memorize the object for 24 h. Alternatively, they may have recognized the object as familiar but needed to examine it for changes after such a long period of time. The fact that the juvenile mice examined the objects more frequently and for longer than the adult mice suggests that this behavior is due to an increased exploratory drive in young animals. A study comparing different mouse strains at 4 months of age found a significant decline in performance from 1 h to 24 h retention time, with some strains not discriminating novel and familiar objects 24 h after their first encounter (53). Accordingly, by choosing a retention time of 24 h, we set a high threshold that requires a good long-term memory. Perhaps we would not have found any differences between the mouse lines with a retention time of 1 h, but we would have possibly found object recognition after 1 h in the adolescent mice. Ideally, we should have tested different inter-test intervals but this would have required more mice. Future studies should be performed with a shorter inter-test interval.

Activity levels in the open arms of the EPM are commonly used as an indicator of individual anxiety. As the DU6 mice generally showed less activity than the FztDU mice, we had to take this into account in our analyses. In fact, the DU6 mice entered the open arms less frequently than the FztDU mice, but they also entered the closed arms less often than the FztDU mice. Consequently, anxiety behavior can only be assessed by calculating the percentage of entries into the open arms relative to all arm entries. The same applies to the distances traveled within the open arms and the time spent in the open arms. We conclude from these relative values that the DU6 mice were not more anxious than the FztDU mice. The behaviors resulting from the conflict of exploring unknown territory, such as the open arms while avoiding open terrain, measured here as head dips into the open arms and the percentage of time spent in the central position, can be interpreted as behaviors of increased caution (40). In our study, DU6 mice exhibit reduced head dips and a lower percentage of time in the central position compared to FztDU mice. However, DU6 mice left the closed arms less frequently than FztDU mice, meaning that they did not pass the central position as often as FztDU mice. This reduced approach/avoidance conflict behavior is more likely evidence of the generally lower activity of the DU6 mice than of reduced caution.

We also learned that the size of the maze in relation to body size has an influence on anxiety-related behavior from our experiment with different-sized EPM devices. An EPM with relatively wide-open arms in relation to the body size of the mice seems to pose a less perceived danger. At least the risk of falling is markedly lower with wider arms. In other studies, the same EPM was used for normal and obese mice. Andre et al. used an 8 cm wide EPM for mice fed either with standard chow or a Western diet (28). Dinel et al. used an 8 cm wide EPM for *db*/*db* and *db*/+ mice (27). Nevertheless, the sizes of obese and control mice in these studies did not differ as much as they did in our study (27, 28).

In general, reduced locomotor activity in exploratory tasks could be misinterpreted as increased anxiety or impaired memory. Size and weight differences complicate the comparison of mouse lines. Therefore, it is crucial to consider all parameters that can be influenced by reduced locomotor activity. In our experiments, we found reduced locomotor activity in DU6 mice. Nonetheless, by interpreting the EPM data based on the relative time spent in the open arms and the relative number of entries into the open arms in relation to entries into open and closed arms, we can distinguish whether only activity or anxiety is affected. In the NOR task, DU6 mice showed a longer duration of inactivity but no difference in running time compared to FztDU mice. When exploring the familiar or novel object, there were no significant differences in exploration of the novel object, indicating that the lack of discrimination between the novel and familiar objects cannot be attributed to reduced locomotor activity.

Obesity is generally characterized by a significant infiltration of adipose tissue by inflammatory cells, contributing to a low-grade systemic inflammatory state (54, 55). This condition is characterized by the excessive secretion of proinflammatory cytokines into the circulation (55), coupled with a reduction in the synthesis of anti-inflammatory adipokines, such as adiponectin. This imbalance fosters a proinflammatory environment, which is further exacerbated by the recruitment of macrophages that amplify the release of cytokines such as TNF-α, IL-6, and IL-1β. In contrast, we did not detect any of the proinflammatory cytokines TNF-α, IL-6 or IL-1β in the serum of seven-week-old DU6 mice. This is in contrast to the findings of Müller-Eigner and colleagues (31), who reported elevated plasma levels of TNF-α and IL-6 in DU6 mice at 16 to 17 weeks of age using an electrochemiluminescence multiplexed assay system. This contradiction can be explained by the fact that the increased inflammation values may manifest in older mice. Moreover, the TNF-α values reported by Müller-Eigner and colleagues (31) are not significantly above the detection limit of the ELISA that we used. A study in *db*/*db* mice at 10-12 weeks of age showed that the values in DU6 mice are not extremely high compared to other obesity models, with TNF-α values approximately twice as high and IL-6 concentrations more than three times higher (27). The study in *db*/*db* mice also showed significantly increased IL-1β concentrations, well above the detection limit of our ELISA (27). However, our transcriptomic analyses of abdominal fat and brain tissue also revealed no evidence of systemic inflammation or neuroinflammation in DU6 mice.

According to Müller-Eigner and colleagues, DU6 mice do not exhibit metabolically healthy obesity, as they have high triglyceride levels, high LDL and HDL cholesterol levels, poor insulin regulation, and low chronic inflammation, along with a greatly reduced life expectancy (31). Strikingly, the DU6 mouse line does not develop diabetes. Of note, fasting glucose levels are not elevated; however, the DU6 mice exhibit impaired glucose clearance in a glucose tolerance test at 10-11 weeks of age, but not at 19-20 weeks of age. This suggests a counter-regulatory mechanism that protects DU6 mice from developing diabetes. The underlying mechanisms of this phenomenon are still elusive. Previous research on the *ob/ob* mouse line (25) postulated that upregulation of MDSCs serves as a mechanism for suppressing the typical obesity-induced low-grade inflammation, which may provide insights into the unique characteristics of the DU6 model. We detected markedly increased numbers of MDSCs expressing the typical monocyte/macrophage marker CD11b (encoded by the *Itgam* gene) and the granulocyte marker Gr-1 (gamma response protein 1 encoded by *Ly6g*) in the spleens of DU6 mice. This heterogeneous MDSC population has long been recognized for its immunosuppressive functions (56, 57) and displayed a crucial role in maintaining immune homeostasis in obese rodents (25). Their concentration in the murine spleen is typically low, ranging between 2 and 4% (58). Notably, MDSCs exhibit minimal expression of MHC class II molecules for antigen presentation and lack co-stimulatory signals, preventing them from activating T cells. Instead, MDSCs suppress CD8^+^ T-cell responses and promote the development of regulatory T cells (56, 59, 60). This immunosuppressive role may offer protection against diabetes. Consistent with this understanding, we persistently detected reduced T cell numbers in obese DU6 mice, accompanied by diminished T-cell activation in response to the mitogen ConA. The lack of systemic inflammation as judged from the absence of classic inflammatory markers TNF-α, IL-1β or IL6 in the periphery of DU6 mice in our study may reflect the efficient immunosuppression and probably underpins the resistance of DU6 mice to diabetes. Admittedly, we have not directly tested the anti-inflammatory function of MDSC in our mouse model by functional tests of cytokine secretion by T cells or of suppression of CD8+ T cell function other than *in vitro* activation-marker expression. Future studies should include such functional tests. Given that inflammation is a key driver of obesity-related diabetes, the lack of both inflammation and diabetes in DU6 mice suggests the immune system plays a pivotal role in mediating this resistance.

Xia et al. demonstrated that MDSCs are increased in diabetic *ob*/*ob* mice (25). Strikingly, wild type mice fed a high-fat diet showed increased numbers of MDSCs. Individuals with depleted MDSCs suffered from more pronounced diabetes, while individuals with enriched MDSCs had less severe diabetes symptoms, leading to the conclusion that this cell population may protect against diabetes (25, 61). Increased numbers of MDSCs have also been found in humans with suppressive effects on inflammation (59, 62).

The differences in leukocyte populations in FztDU and DU6 mice were not limited to MDSCs. DU6 mice also had a higher proportion of B cells and a lower proportion of T cells than FztDU mice and a reduced T-cell activation. These alterations may be associated with increased numbers of MDSCs, which are known to suppress T cell responses (25).

Our previous studies (40, 41) demonstrated that the EPM acts as a moderate psychological stressor for mice, leading to increased plasma corticosterone concentrations. In the present study, we also observed that the corticosterone levels in EPM-challenged FztDU mice rose to match those in DU6 mice, where corticosterone concentrations appear to be permanently elevated. Although elevated glucocorticoid levels are usually associated with higher anxiety (63), we did not find increased anxiety in DU6 mice. Nevertheless, in other rodent models of obesity, corticosterone and anxiety were elevated in obese mice (27, 28). It is therefore unlikely that glucocorticoids are directly responsible for increased anxiety in obesity.

Transcriptome analysis of the hippocampi from both mouse lines identified three distinctly differentially regulated genes. In DU6 mice, the *Mid1* gene (alias *Rnf59* and *Trim18*), which encodes midline protein-1, was significantly upregulated. Given that all the mice sampled were male, this expression difference is unlikely due to sex-based variability, as previously observed in a microarray study in mice (64). *Mid1* is a microtubule-associated protein (65) critical for cell cycle dynamics and stability (66). It is highly expressed in murine microglia (67). The knockout of Mid1 in murine macrophages elevated the levels of type I interferons upon stimulation with virus or viral PAMPs, without affecting the expression of CD11b on macrophages (67). In line with this, interferon signaling has been identified as a canonical pathway differentially regulated in the hippocampi of DU6 and FztDU mice. In human patients with diabetic kidney disease, renal MID1 levels were increased compared to controls. These *MID1* levels correlated with the signaling of STAT3, which is induced by interferons (68). Additionally, Mid1’s interaction with microtubules may influence the mammalian targets of rapamycin complex 1 (mTORC1) signaling (69). It is worth noting that whole-genome sequencing identified a single nucleotide polymorphism (SNP) in *Mid1-ps1* (midline 1 pseudogene 1) on the Y chromosome of mice with polygenic obesity (fat line) compared to lean individuals (70). However, this SNP provides limited insight into why *Mid1* is so strongly expressed in the hippocampus of DU6 mice.

*Gjb4* encodes gap junction protein beta 4 and revealed, similar to *Mid1*, strongly increased transcript levels in DU6 mice compared with FztDU mice. Members of the Gjb family facilitate the transport of glucose and lactate between astrocytes and neurons and were previously found to be downregulated in the hippocampi of non-obese type 2 diabetes rats compared to healthy controls (71), while elevated *Gjb4* levels in the islet cells of an obese mouse strain with permanently increased blood-glucose levels were demonstrated to contribute to the pathogenesis of diabetes (72).

In FztDU mice, the expression of the *Ccl19* gene, encoding C-C motif chemokine ligand 19 (also known as macrophage inflammatory protein 3-beta), was dominant. *Ccl19* is a recognized marker of numerous inflammatory conditions, including the chronic low-grade inflammation associated with obesity and diabetic nephropathy (73). Our transcriptomic analyses revealed that the expression of *Ccl19* in the hippocampus, hypothalamus, and abdominal adipose tissue is lower in obese DU6 mice than in control FztDU mice. Since *Ccl19* transcript levels have been demonstrated to be elevated in adipose tissue of obese individuals with or without diabetes (74, 75), it is surprising that the *Ccl19* concentration is comparatively low in obese DU6 mice. The expression of *Ccl19* is not restricted to one specific immune cell population but has been identified in a variety of cells, including macrophages, dendritic cells, fibroblastic reticular cells, lymphatic endothelial cells, or stromal cells (76–79). Although elevated *CCL19* levels are associated with neuroinflammatory diseases (80–82), it remains unclear whether the low *Ccl19* expression observed in the present study provides an explanation for the absence of neuroinflammation in DU6 mice despite obesity. Whether the low *Ccl19* expression in DU6 mice is related to the higher number of immunosuppressive MDSCs needs to be clarified.

To rule out neuroinflammation as the cause of cognitive deficits in DU6 mice, we specifically searched our microarray data from the hippocampus for pro- and anti-inflammatory cytokines and glial activation. The data show no differences between the mouse lines in either glial activation or cytokine expression. Anti-inflammatory cytokines could also have played a role here. For example, IL-10 blocks the inhibitory effect of IL-1β on long-term potentiation, which is crucial for learning (83). Microglial TGF-β is necessary for maintaining cognitive functions (84).

In addition to changes in the transcriptome, epigenetic modifications such as chromatin remodeling and DNA methylation could also be relevant. Epigenetic studies conducted on liver tissue from male DU6 mice have revealed altered histone acetylation levels, which may contribute to changes in energy metabolism (31). This aligns with the fact that DU6 mice are known to have a higher feed intake combined with better feed conversion, which is reflected in their energy metabolism (35). It would be interesting to also investigate potential epigenetic alterations in the hypothalamus, as this brain region regulates appetite and food intake. In a recent study, an altered DNA methylation profile was identified in the hypothalamus of male 5xFAD mice, an age-related Alzheimer’s-like model. Among other pathways, the oxytocin (OXT) and gonadotropin-releasing hormone (GnRH) signaling pathways were affected. This profile appears to underlie deficits in object memory in the absence of inflammation, as treatment with OXT and GnRH counteracted the cognitive deficits (85). Oxytocin also plays a role in food intake and energy metabolism (88). Given that DU6 mice exhibit persistently elevated stress hormone levels, it may be worthwhile to investigate whether the DU6 phenotypes are due to neuroendocrine dysfunction rather than systemic inflammation or neuroinflammation.

To summarize, we found that DU6 mice show neither systemic inflammation nor neuroinflammation, no increased anxiety-like behavior, and no spatial memory deficits but rather a dysregulation of the HPA axis and impaired object memory. The increased number of myeloid-derived suppressor cells may be responsible for suppressing inflammation and providing protection against diabetes. The lower numbers of T cells and decreased T-cell activation illustrate the significant impact of immune regulation on metabolic health. It is also possible that the reduced number of T cells may contribute to object memory impairment, as T cells have been found to play a key role in regulating cognition (for review see (86)). Our study provides evidence for altered hypothalamic feedback suggesting a mechanistic link between neuroendocrine regulation, cognitive behavior and immunometabolic responses that should be addressed in future studies. These studies need to investigate the effects of MDSC-mediated suppression of T-cell function on metabolic health and cognitive function to better understand the health consequences associated with obesity. A limitation of this study is the exclusive use of male mice. The prevalence of obesity is higher among women than men worldwide, although in many regions the opposite is true (87). However, future studies should also include female animals to determine whether the observed effects are consistent in both sexes.

## Data Availability

The datasets presented in this study can be found in online repositories. The names of the repository/repositories and accession number(s) can be found below: https://www.ncbi.nlm.nih.gov/geo/, GSE280980.

## Supplementary material

Supplementary Figure 1 Exploration behavior of mature adult and adolescent male FztDU and DU6 mice toward novel and familiar objects. Panels **(A–D)** show the number of contacts with familiar (black bars) and novel (gray bars) objects in each minute over a 10-minute period. Panels **(E–H)** display the duration of exploration per contact [s]. Data are presented separately for mature adult **(A, B, E, F)** and adolescent **(C, D, G, H)** mice from the FztDU (green label) and DU6 (orange label) lines. Values represent LS means ± SE.

Supplementary Figure 2 Ability to distinguish between new and familiar objects in adult male FztDU and DU6 mice and male juvenile FztDU and DU6 mice in the novel object recognition test.
