# Supplementary material for: A unique polygenic mouse model of obesity exhibits a distinct immunological profile that may offer protection against systemic inflammation, diabetes, and behavioral impairments
Source: Front Immunol. 2025 Sep 12;16:1601809. doi: 10.3389/fimmu.2025.1601809 (PMC12504882; doi:10.3389/fimmu.2025.1601809)
Supplement: Supplementary Figure 1 — Exploration behavior of mature adult and adolescent male FztDU and DU6 mice toward novel and familiar objects. Panels (A–D) show the number of contacts with familiar (black bars) and novel (gray bars) objects in each minute over a 10-minute period. Panels (E–H) display the duration of exploration per contact [s]. Data are presented separately for mature adult (A, B, E, F) and adolescent (C, D, G, H) mice from the FztDU (green label) and DU6 (orange label) lines. Values represent LS means ± SE. [file Image1.pdf]

frontiers

Supplementary Material

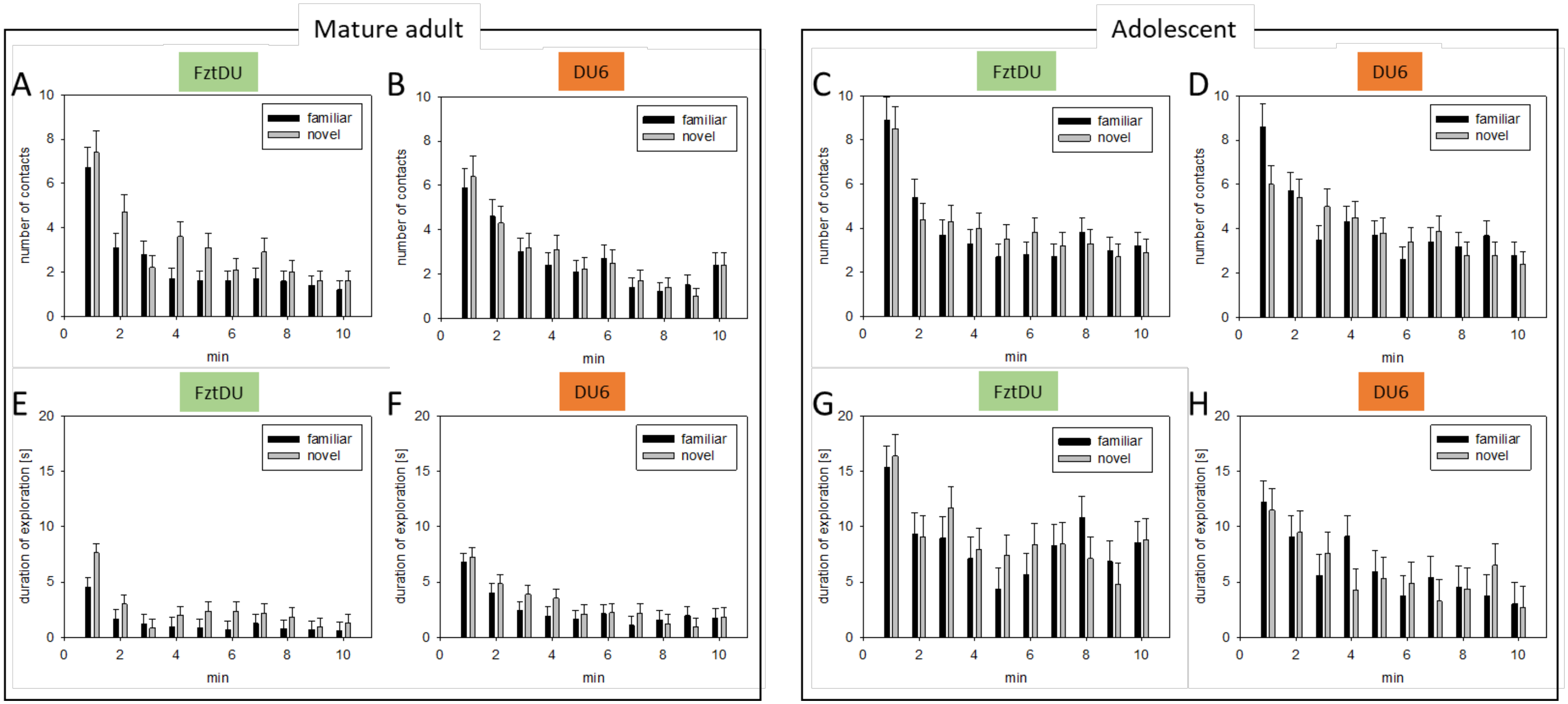

Supplementary Figure S1: Exploration behavior of mature adult and adolescent male FztDU and DU6 mice toward novel and familiar objects. Panels (A–D) show the number of contacts with familiar (black bars) and novel (gray bars) objects in each minute over a 10-minute period. Panels (E–H) display the cumulative duration of exploration [s]. Data are presented separately for mature adult (A, B, E, F) and adolescent (C, D, G, H) mice from the FztDU (green label) and DU6 (orange label) lines. Values represent LS means ± SEM.
