# Supplementary material for: A unique polygenic mouse model of obesity exhibits a distinct immunological profile that may offer protection against systemic inflammation, diabetes, and behavioral impairments
Source: Front Immunol. 2025 Sep 12;16:1601809. doi: 10.3389/fimmu.2025.1601809 (PMC12504882; doi:10.3389/fimmu.2025.1601809)
Supplement: Supplementary Figure 2 — Ability to distinguish between new and familiar objects in adult male FztDU and DU6 mice and male juvenile FztDU and DU6 mice in the novel object recognition test. [file Image2.pdf]

Supplementary Material

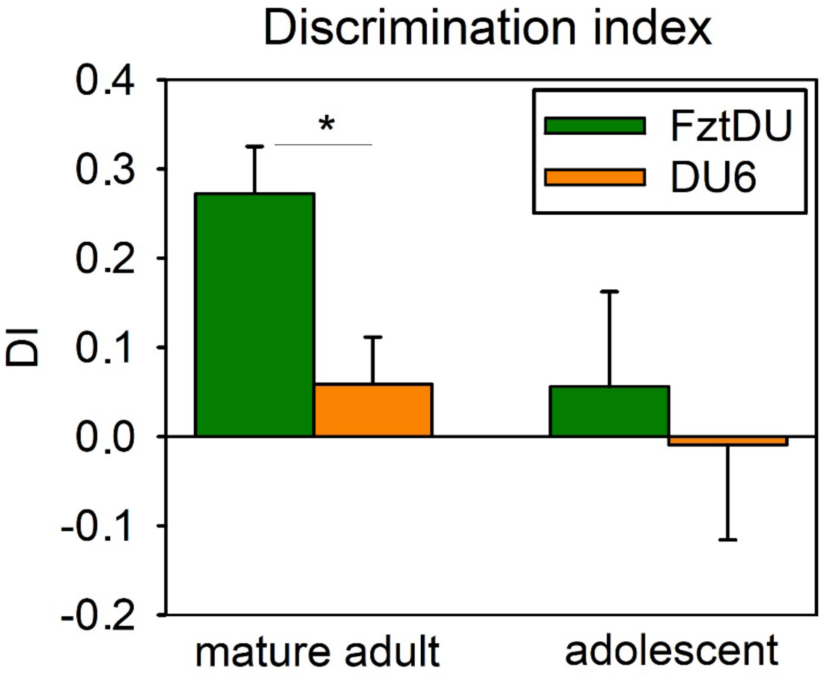

Supplementary Figure S2: Ability to distinguish between new and familiar objects in adult male FztDU and DU6 mice and male juvenile FztDU and DU6 mice in the novel object recognition test. The discrimination index DI was calculated by

$$DI = (t_n - t_f)/(t_n + t_f)$$

with t_n, time spent exploring the novel object and t_f, time spent exploring the familiar object. Data are presented as LS means + SE. Statistical significance is indicated as follows: * $p < 0.05$ (Tukey-Kramer test).
