# Supplementary material for: A unique polygenic mouse model of obesity exhibits a distinct immunological profile that may offer protection against systemic inflammation, diabetes, and behavioral impairments
Source: Front Immunol. 2025 Sep 12;16:1601809. doi: 10.3389/fimmu.2025.1601809 (PMC12504882; doi:10.3389/fimmu.2025.1601809)
Supplement: Supplementary file 3 [file Table1.docx]

Supplementary Material

# Supplementary Table S1. List of antibodies used for flow cytometry with ordering details (all from BioLegend, Amsterdam, Netherlands).

| **Targeted antigen** | **Antibody conjugated with** | **Clone** | **Catalog no.** | **Isotype** | **Clone of matching isotype control** | **Catalog no.** |
| --- | --- | --- | --- | --- | --- | --- |
| CD11b | PE | M1/70 | 101207 | Rat IgG2b, κ | RTK4530 | 400607 |
| Gr-1 (Ly-6/Ly-5) | APC | RB6-8C5 | 108411 | Rat IgG2b, κ | RTK4530 | 400611 |
| CD45.2 | FITC | 104 | 109805 | Mouse IgG2a, κ |  |  |
| CD3 | APC | 145-2C11 | 100311 | Armenian hamster IgG | HTK888 | 400911 |
| CD4 | FITC | GK1.5 | 100405 | Rat IgG2b, κ | RTK4530 | 400605 |
| CD8 | BV421 | 53-6.7 | 100753 | Rat IgG2a | RTK2758 | 400549 |
| B220 | PE/Cy7 | RA3-6B2 | 103221 | Rat IgG2a | RTK2758 | 400521 |
| CD25 | PE/Cy7 | PC61 | 102015 | Rat IgG1, λ | G0114F7 | 401907 |
| CD69 | PE | H1.2F3 | 104507 | Armenian hamster IgG | HTK888 | 400907 |

APC, allophycocyanine; BV421, brilliant violet 421; FITC, fluorescein isothiocyanate; PE, phycoerythrin; Cy7, cyanine 7
