# Supplementary material for: A unique polygenic mouse model of obesity exhibits a distinct immunological profile that may offer protection against systemic inflammation, diabetes, and behavioral impairments
Source: Front Immunol. 2025 Sep 12;16:1601809. doi: 10.3389/fimmu.2025.1601809 (PMC12504882; doi:10.3389/fimmu.2025.1601809)
Supplement: Supplementary file 4 [file Table2.docx]

Supplementary Material

**Supplementary Table** **S2**: **Y-maze behavior of male FztDU and DU6 mice. Mature adult FztDU mice were tested in the standard-sized and wider mazes. Data are shown as LS means ± SE.**

| **Behavior** | **FztDU**  **(standard-sized maze)** | **FztDU**  **(wider maze)** | **DU6**  **(wider maze)** | **p-value (Tukey-Kramer test)**  **FztDU (standard-sized maze) vs. FztDU (wider maze)** |
| --- | --- | --- | --- | --- |
| **Mature adult mice** | **n = 5** | **n = 5** | **n = 10** |  |
| Familiar arm entries (divided by two) | 8.80 ± 1.14 | 12.80 ± 1.38 | 7.90 ± 0.87 | 0.057 |
| Novel arm entries | 11.20 ± 1.29 | 14.60 ± 1.47 | 9.60 ± 0.96 | 0.121 |
| Duration of exploration of familiar arms (s) | 89.39 ± 9.28 | 84.69 ± 9.28 | 87.31 ± 5.81 | 0.983 |
| Duration of exploration of novel arms (s) | 121.22 ± 9.28 | 130.62 ± 9.28 | 125.37 ± 5.81 | 0.888 |
| **Adolescent mice** | **n = 10** |  | **n = 10** |  |
| Familiar arm entries (divided by two) | 9.10 ± 0.61 |  | 6.90 ± 0.53 |  |
| Novel arm entries | 12.10 ± 0.70 |  | 8.40 ± 0.59 |  |
| Duration of exploration of familiar arms (s) | 84.30 ± 5.74 |  | 86.41 ± 5.74 |  |
| Duration of exploration of novel arms (s) | 131.40 ± 5.74 |  | 127.17 ± 5.74 |  |
