# Supplementary material for: A unique polygenic mouse model of obesity exhibits a distinct immunological profile that may offer protection against systemic inflammation, diabetes, and behavioral impairments
Source: Front Immunol. 2025 Sep 12;16:1601809. doi: 10.3389/fimmu.2025.1601809 (PMC12504882; doi:10.3389/fimmu.2025.1601809)
Supplement: Supplementary file 5 [file Table3.docx]

Supplementary Material

# Supplementary Table S3. Flow cytometric characterization of spleen cell populations of male control mice at 7 and 21 weeks of age

| **Surface markers of leukocyte population** | **FztDU**  **7 weeks** | **FztDU**  **21 weeks** | **p-value; FztDU;**  **7 vs. 21 weeks** | **DU6**  **7 weeks** | **DU6**  **21 weeks** | **p-value; DU6;**  **7 vs.**  **21 weeks** |
| --- | --- | --- | --- | --- | --- | --- |
| Gr-1^+^CD11b^+^ | 3.9 ± 0.5% | 3.1 ± 0.8% | 0.839 | 10.0 ± 2.9% | 20.6 ± 5.0% | 0.292 |
| Gr-1^-^CD11b^+^ | 4.6 ± 0.3% | 2.3 ± 0.4% | **< 0.001** | 7.7 ± 0.3% | 3.1 ± 0.4% | **< 0.001** |
| B220^+^ | 54.0 ± 1.0% | 48.5 ± 1.7% | **< 0.05** | 56.6 ± 2.1% | 54.2 ± 3.7% | 0.943 |
| CD3^+^ | 30.5 ± 1.1% | 34.4 ± 1.9% | 0.100 | 11.3 ± 0.9% | 12.3 ± 1.6% | 0.948 |
| CD4^+^CD3^+^ | 23.1 ± 1.3% | 27.8 ± 2.3% | 0.295 | 8.8 ± 0.9% | 11.4 ± 1.6% | 0.492 |
| CD8^+^CD3^+^ | 9.4 ± 0.6% | 9.5 ± 1.0% | 1.000 | 3.5 ± 0.4% | 3.3 ± 0.6% | 0.991 |

Results are presented as LS means ± SE, and the p-values of the Tukey-Kramer test; n=15 per mouse line at 7 weeks of age; n=5 per mouse line at 21 weeks of age.
