# Supplementary material for: A unique polygenic mouse model of obesity exhibits a distinct immunological profile that may offer protection against systemic inflammation, diabetes, and behavioral impairments
Source: Front Immunol. 2025 Sep 12;16:1601809. doi: 10.3389/fimmu.2025.1601809 (PMC12504882; doi:10.3389/fimmu.2025.1601809)
Supplement: Supplementary file 6 [file Table4.docx]

Supplementary Material

# Supplementary Table S4. Flow cytometric characterization of T cell activation in response to ConA stimulation.

| **Surface markers of leukocyte population** | **FztDU**  **control** | **DU6**  **control** | **p-value; FztDU vs. DU6**  **(control)** | **FztDU**  **stress** | **DU6**  **stress** | **p-value; FztDU vs. DU6**  **(stress)** | **p-value; FztDU control vs. stress** | **p-value; DU6 control vs. stress** |
| --- | --- | --- | --- | --- | --- | --- | --- | --- |
| CD25^+^CD4^+^ | 15.7 ± 0.9 | 6.0 ± 0.5 | **< 0.001** | 22.7 ± 1.1 | 8.5 ± 0.7 | **< 0.001** | **< 0.001** | **< 0.05** |
| CD69^+^CD4^+^ | 21.4 ± 1.1 | 7.3 ± 0.6 | **< 0.001** | 28.2 ± 1.3 | 8.7 ± 0.8 | **< 0.001** | **< 0.01** | 0.497 |
| CD25^+^CD8^+^ | 10.5 ± 0.8 | 4.1 ± 0.3 | **< 0.001** | 14.3 ± 1.0 | 4.1 ± 0.4 | **< 0.001** | **< 0.05** | 1.00 |
| CD69^+^CD8^+^ | 16.8 ± 1.1 | 5.2 ± 0.5 | **< 0.001** | 15.1 ± 1.4 | 4.3 ± 0.6 | **< 0.001** | 0.794 | 0.677 |
|  |  |  |  |  |  |  |  |  |
| CD25^-^CD4^+^ | 10.3 ± 1.0 | 4.7 ± 0.3 | **< 0.001** | 14.3 ± 1.3 | 5.8 ± 0.3 | **< 0.001** | 0.103 | **< 0.05** |
| CD69^-^CD4^+^ | 3.7 ± 0.6 | 4.0 ± 0.4 | 0.979 | 7.4 ± 0.7 | 4.2 ± 0.5 | **< 0.01** | **< 0.01** | 0.987 |
| CD25^-^CD8^+^ | 13.2 ± 0.7 | 5.2 ± 0.3 | **< 0.001** | 17.5 ± 0.8 | 3.0 ± 0.4 | **< 0.001** | **< 0.01** | **< 0.01** |
| CD69^-^CD8^+^ | 3.6 ± 0.3 | 3.5 ± 0.2 | 1.0 | 3.6 ± 0.4 | 1.8 ± 0.2 | **< 0.001** | 1.00 | **< 0.001** |
|  |  |  |  |  |  |  |  |  |
| CD25^+^CD4^+^/CD4^+^ | 62.8 ± 1.8% | 54.9 ± 1.5% | **< 0.05** | 62.3 ± 2.3% | 57.8 ± 1.9% | 0.425 | 0.998 | 0.626 |
| CD69^+^CD4^+^/CD4^+^ | 85.7 ± 1.4% | 63.7 ± 2.4% | **< 0.001** | 79.9 ± 1.7% | 65.9 ± 2.9% | **< 0.01** | 0.063 | 0.930 |
| CD25^+^CD8^+^/CD8^+^ | 41.7 ± 1.6% | 43.6 ± 1.4% | 0.806 | 47.8 ± 2.0% | 56.4 ± 1.7% | **< 0.01** | 0.090 | **< 0.001** |
| CD69^+^CD8^+^/CD8^+^ | 81.7 ± 1.9% | 58.0 ± 1.5% | **< 0.001** | 80.0 ± 2.4% | 69.8 ± 1.9% | **< 0.01** | 0.944 | **< 0.001** |

Results are presented as LS means ± SE and the p-values of the Tukey-Kramer test; n=15 male mice per mouse line in control group; n=10 male mice per mouse line in stress group at 7 weeks of age.
