# Supplementary material for: A unique polygenic mouse model of obesity exhibits a distinct immunological profile that may offer protection against systemic inflammation, diabetes, and behavioral impairments
Source: Front Immunol. 2025 Sep 12;16:1601809. doi: 10.3389/fimmu.2025.1601809 (PMC12504882; doi:10.3389/fimmu.2025.1601809)
Supplement: Supplementary file 7 [file Table5.docx]

Supplementary Material

# Supplementary Table S5. Selected microarray-based gene expression data comparing DU6 and FztDU mice (n = 5).

| **Gene symbol** | **Protein encoded** | **Array ID**  **(suffix ‛.mm.2’)** | **DU6 (1)** | **DU6 (2)** | **DU6 (3)** | **DU6 (4)** | **DU6 (5)** | **FztDU (1)** | **FztDU (2)** | **FztDU (3)** | **FztDU (4)** | **FztDU (5)** | **Du6 Avg (log2)** | **FztDu Avg (log2)** | **Fold Change DU6/FztDu** | **P-val** | **FDR P-val** |
| --- | --- | --- | --- | --- | --- | --- | --- | --- | --- | --- | --- | --- | --- | --- | --- | --- | --- |
| ***Cd11b/***  ***Itgam*** | Integrin alpha M | TC0700001875 | 6.46 | 5.76 | 6.79 | 6.53 | 6.59 | 6.95 | 6.77 | 6.95 | 6.81 | 6.82 | 7.75 | 8.33 | -1.50 | 0.01 | 0.52 |
| ***Cd68*** | CD68 antigen | TC1100003055 | 6.22 | 6.02 | 6.34 | 6.16 | 6.50 | 6.08 | 5.88 | 5.93 | 5.83 | 6.25 | 7.74 | 7.25 | 1.40 | 0.04 | 0.71 |
| ***Gfap*** | Glial fibrillary acidic protein | TC1100003856 | 8.81 | 8.91 | 8.89 | 9.10 | 8.88 | 9.09 | 8.68 | 8.87 | 9.13 | 8.61 | 12.75 | 12.75 | -1.00 | 0.76 | 0.98 |
| ***Il1b*** | Interleukin 1 beta | TC0200004602 | 3.01 | 2.99 | 2.62 | 2.98 | 3.06 | 2.72 | 3.00 | 2.59 | 2.71 | 3.00 | 4.58 | 4.24 | 1.27 | 0.25 | 0.91 |
| ***Il4*** | Interleukin-4 | TC0100001167 | 2.79 | 2.33 | 2.79 | 2.92 | 2.84 | 2.63 | 3.10 | 2.99 | 2.78 | 2.76 | 4.25 | 4.16 | 1.06 | 0.61 | 0.96 |
| ***Il6*** | Interleukin-6 | TC0500000283 | 1.89 | 2.03 | 1.59 | 1.99 | 1.89 | 2.31 | 1.96 | 2.09 | 2.04 | 1.86 | 2.74 | 2.85 | -1.08 | 0.18 | 0.88 |
| ***Il10*** | Interleukin-10 | TC1100002737 | 2.88 | 2.95 | 2.97 | 2.98 | 3.50 | 2.67 | 3.37 | 2.86 | 2.76 | 2.98 | 2.81 | 2.73 | 1.05 | 0.29 | 0.92 |
| ***Il13*** | Interleukin-13 | TC1100002738 | 5.10 | 4.83 | 4.62 | 5.14 | 5.49 | 4.91 | 4.76 | 4.70 | 5.29 | 5.12 | 3.28 | 3.20 | 1.05 | 0.52 | 0.95 |
| ***Mc4r*** | Melanocortin 4 receptor | TC1800001485 | 4.84 | 4.62 | 4.40 | 5.14 | 4.62 | 5.63 | 5.01 | 5.29 | 5.06 | 5.33 | 4.87 | 5.74 | -1.83 | 0.00 | 0.31 |
| ***Nr3c1*** | Nuclear receptor subfamily 3. group C. member 1 | TC1800001243 | 6.47 | 6.72 | 7.00 | 6.52 | 6.47 | 6.59 | 6.71 | 6.72 | 6.27 | 6.66 | 8.15 | 8.18 | -1.02 | 0.53 | 0.96 |
| ***Tgfb*** | Transforming growth factor, beta 1 | TC0700000424 | 5.62 | 5.61 | 5.76 | 5.80 | 5.64 | 5.88 | 5.62 | 5.78 | 5.60 | 5.76 | 7.43 | 7.44 | -1.00 | 0.77 | 0.98 |
| ***Tnf*** | Tumor necrosis factor | TC1700001954 | 2.57 | 2.82 | 2.73 | 2.76 | 3.13 | 2.73 | 3.21 | 2.64 | 2.78 | 2.47 | 2.56 | 2.48 | 1.06 | 0.63 | 0.96 |
